# How Lewis Acids Catalyze Diels–Alder Reactions

**DOI:** 10.1002/anie.201914582

**Published:** 2020-02-19

**Authors:** Pascal Vermeeren, Trevor A. Hamlin, Israel Fernández, F. Matthias Bickelhaupt

**Affiliations:** ^1^ Department of Theoretical Chemistry Amsterdam Institute of Molecular and Life Sciences (AIMMS) Amsterdam Center for Multiscale Modeling (ACMM) Vrije Universiteit Amsterdam De Boelelaan 1083 1081 HV Amsterdam The Netherlands; ^2^ Departamento de Química Orgánica I and Centro de Innovación en Química Avanzada (ORFEO-CINQA) Facultad de Ciencias Químicas Universidad Complutense de Madrid 28040 Madrid Spain; ^3^ Institute for Molecules and Materials (IMM) Radboud University Heyendaalseweg 135 6525 AJ Nijmegen The Netherlands

**Keywords:** Activation strain model, density functional calculations, Diels–Alder reactions, Lewis acid catalysis, Pauli repulsion

## Abstract

The Lewis acid(LA)‐catalyzed Diels–Alder reaction between isoprene and methyl acrylate was investigated quantum chemically using a combined density functional theory and coupled‐cluster theory approach. Computed activation energies systematically decrease as the strength of the LA increases along the series I_2_<SnCl_4_<TiCl_4_<ZnCl_2_<BF_3_<AlCl_3_. Emerging from our activation strain and Kohn–Sham molecular orbital bonding analysis was an unprecedented finding, namely that the LAs accelerate the Diels–Alder reaction by a diminished Pauli repulsion between the π‐electron systems of the diene and dienophile. Our results oppose the widely accepted view that LAs catalyze the Diels–Alder reaction by enhancing the donor–acceptor [HOMO_diene_–LUMO_dienophile_] interaction and constitute a novel physical mechanism for this indispensable textbook organic reaction.

## Graphical abstract

**Expect the unexpected**! Quantum chemical activation strain analyses reveal that Lewis acids accelerate Diels–Alder reactions by reducing the Pauli repulsion between the reactants. This novel physical mechanism for the textbook Diels–Alder reaction challenges the wide‐held belief that the Lewis acids lower the LUMO energy of the dienophile and, in that way, make them more reactive toward the diene.

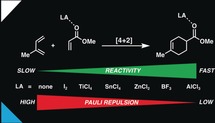

Nearly one century after the discovery of the Diels–Alder reaction by O. Diels and K. Alder in 1928,^1^ this transformation is still one of the most relevant processes in chemistry.^2^ This is mainly due to the fact that this reaction is able to produce six‐membered rings, generating up to four stereocenters in a single reaction step, and, therefore, significantly increasing the molecular complexity. For this reason, this particular transformation has been widely applied towards the preparation of a huge number of target compounds, including complex natural products as well as systems with potential applications in medicinal chemistry or materials science.^3^ The potential of this particular reaction is also acknowledged in industry because it allows for the rapid formation of complex structures whilst fulfilling atom‐economy criteria.^4^

It is well known that Diels–Alder reactions are greatly accelerated by Lewis acids (LAs) via complexation to the dienophile.^5^ These LA‐catalyzed cycloadditions are not only faster than their uncatalyzed analogues, but are also generally more regio‐ and stereoselective. According to frontier molecular orbital (FMO) theory and a plethora of mechanistic studies on these reactions,^6^ it is nowadays widely accepted that the donor–acceptor interaction established between the dienophile and the LA catalyst results in a significant stabilization of the LUMO of the dienophile, which is ultimately translated into a smaller HOMO_diene_–LUMO_dienophile_ energy gap and, as a consequence, to a lower reaction barrier as compared to the uncatalyzed reaction.^7^ A similar result was found by us quite recently when studying, amongst others, the catalytic ability of dihalogen catalysts (X_2_=F_2_ to I_2_) on the Michael addition reaction.^8^ However, although these species also lower the energy of the LUMO of the Michael acceptor, the total orbital interactions between the reagents along the reaction coordinate (including the key HOMO–LUMO interaction) are not stronger, but are even slightly weaker than those present in the uncatalyzed reaction. Indeed, we reported that the origin of the catalysis by dihalogen molecules is therefore not ascribed to the strength of the orbital interactions but to a significant decrease of the two‐center four‐electron Pauli repulsion between the lone pair of the nucleophile and the π‐system of the Michael acceptor. This hitherto unexpected electronic mechanism prompted us to hypothesize whether this behavior is not only restricted to these particular X_2_‐catalyzed reactions but general and fully applicable to any LA‐catalyzed reaction. To check the generality of this mode of action, we have selected the textbook Diels–Alder reaction involving isoprene (diene) and methyl acrylate (ester), which, in the presence of LAs such as AlCl_3_, leads to the almost selective formation (95:5) of the corresponding 1,4‐cycloadduct (see Scheme 1).^9^

**Scheme 1:**
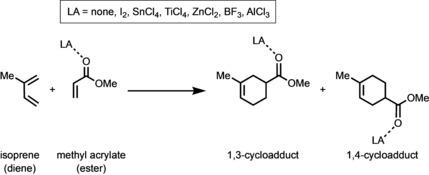
The uncatalyzed and Lewis‐acid(LA)‐catalyzed Diels–Alder reactions between isoprene (diene) and methyl acrylate (ester) that were computationally analyzed.

The nature of the interaction between the LA and methyl acrylate (ester) in the LA–ester complexes was analyzed using the energy decomposition analysis (EDA; see below) method at the ZORA‐BP86/TZ2P level (Table 1).^10^ Similar results were obtained at the dispersion‐corrected ZORA‐BP86‐D3(BJ)/TZ2P level, where the contribution of dispersion to the total interaction energies is only 5–11 % (see Table S2, Supporting Information). Not surprisingly, the interaction energy (Δ*E*_int_) steadily becomes more stabilizing from I_2_ to AlCl_3_ and ranges from −5.5 to −37.5 kcal mol^−1^, which is in line with the relative Lewis acidity of the LA.^11^ The electrostatic interaction (Δ*V*_elstat_) follows the same trend as the interaction energies and is, in all cases, the main contributor to the magnitude of Δ*E*_int_. This confirms the highly polarized nature of the LA⋅⋅⋅O=C bonds. Nevertheless, the orbital interactions (Δ*E*_oi_) are nearly as stabilizing as Δ*V*_elstat_ and are mainly the result of the donor–acceptor, that is, dative, bond established between the lone pair of the carbonyl oxygen atom and the vacant (atomic d or p) orbital of the LA. As expected, Δ*E*_oi_ follows the same trend as Δ*E*_int_, which again agrees with the relative Lewis acidities of the LA species included in this study. Strikingly, although the energy of the LUMO of these LA–ester complexes, which corresponds to the reactive π*‐molecular orbital, is more stable (that is, more negative) than that of the parent methyl acrylate (−2.6 eV), it does not follow the same trend in reactivity (see Table 2). This finding, in principle, suggests that the relative reactivity of these dienophiles is not directly related to the corresponding HOMO_diene_–LUMO_ester_ interaction, as widely accepted.

**Table 1:** Energy decomposition analysis terms (in kcal mol^−1^), LUMO orbital energy, *ϵ*_LUMO_, of the LA–ester complex (in eV), and LA⋅⋅⋅O=C distance (in Å), computed on LA‐ester complexes,^[a]^ as well as experimentally determined relative Lewis acidity.^[b]^ 
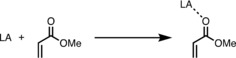

LA	Δ*E*_int_	Δ*V*_elstat_	Δ*E*_Pauli_	Δ*E*_oi_	*ϵ*_LUMO_	*r*(LA⋅⋅⋅O=C)	Relative Lewis acidity^[b]^
I_2_	−5.5	−13.9	18.6	−10.2	−3.7	2.745	–^[c]^
SnCl_4_	−10.0	−31.5	40.8	−19.2	−4.0	2.493	0.52±0.04
TiCl_4_	−14.5	−41.5	50.6	−23.6	−4.3	2.250	0.66±0.03
ZnCl_2_	−17.2	−41.5	46.8	−22.5	−3.7	2.129	–^[c]^
BF_3_	−25.6	−59.8	87.0	−52.8	−3.8	1.733	0.77±0.02
AlCl_3_	−37.5	−65.7	74.4	−46.2	−4.2	1.898	0.82

[a] The Lewis acid (LA) and ester constitute the two interacting fragments. Computed at ZORA‐BP86/TZ2P. [b] Relative Lewis‐acidity scale based on Δδ‐values of H3 resonances of various bases related to methyl crotonate, data taken from ref. 11a. [c] No data available.

**Table 2:** Electronic reaction barriers (Δ*E* ^≠^) and reaction energies (Δ*E*_rxn_) (in kcal mol^−1^) computed for the uncatalyzed and LA‐catalyzed Diels–Alder reaction between isoprene (diene) and methyl acrylate (ester).

LA	cycloadduct	Δ*E* ^≠[a]^	Δ*E* ^≠[b]^	Δ*E* ^≠[c]^	Δ*E* ^≠[d]^	Δ*E*_rxn_^[a]^
none	1,4	13.6	14.8	12.1	16.2	−37.5
	1,3	14.2	15.1	12.4		−37.6
I_2_	1,4	11.0	13.3	9.4	15.0	−14.8
	1,3	11.9	13.7	9.8		−16.1
SnCl_4_	1,4	10.1	10.6	7.2	12.3	−18.1
	1,3	11.2	11.3	7.9		−14.3
TiCl_4_	1,4	8.9	9.1	5.6	11.8	−38.8
	1,3	9.0	9.9	6.3		−36.9
ZnCl_2_	1,4	8.8	8.4	5.6	10.8	−18.9
	1,3	9.7	9.1	6.3		−18.8
BF_3_	1,4	7.7	7.1	4.7	10.0	−39.2
	1,3	8.8	8.2	5.7		−36.4
AlCl_3_	1,4	5.2	5.2	1.6	7.6	−20.4
	1,3	6.4	6.4	2.6		−18.1

[a] Computed at ZORA‐BP86/TZ2P. [b] Computed at ZORA‐M06‐2X‐D3/QZ4P//ZORA‐BP86/TZ2P. [c] Computed at ZORA‐B3LYP‐D3(BJ)/QZ4P//ZORA‐BP86/TZ2P. [d] Computed at (TightPNO)DLPNO‐CCSD(T)/CBS(3,4/def2)//ZORA‐BP86/TZ2P.

The electronic reaction barriers (Δ*E* ^≠^) and reaction energies (Δ*E*_rxn_) of the uncatalyzed and LA‐catalyzed Diels–Alder reaction between isoprene (diene) and methyl acrylate (ester) are provided in Table 2 (see Figure S1 for transition state structures). In every studied reaction, the 1,4‐pathway is kinetically favored over the 1,3‐pathway, which is in line with the well‐established *ortho*–*para* rule^12^ and consistent with the selective formation (95:5) of the corresponding 1,4‐cycloadduct for the reaction involving AlCl_3_ as a catalyst observed experimentally.^9^ As expected, the uncatalyzed reaction has the highest barriers, 13.6 and 14.2 kcal mol^−1^, leading to the 1,4‐ and 1,3‐cycloadducts, respectively, while coordination of a LA to the ester results in lower barrier heights that systematically decrease when going from I_2_ to AlCl_3_, 11.0 and 11.9 kcal mol^−1^ to 5.2 and 6.4 kcal mol^−1^ for the 1,4‐ and 1,3‐adducts, respectively. The computed trends in reactivity at ZORA‐BP86/TZ2P agree well with those calculated at ZORA‐M06‐2X‐D3/QZ4P//ZORA‐BP86/TZ2P, ZORA‐B3LYP‐D3(BJ)/QZ4P//ZORA‐BP86/TZ2P, and the more accurate (TightPNO)DLPNO‐CCSD(T)/CBS(3,4/def2)//ZORA‐BP86/TZ2P level, as well as with the experimentally determined11a relative acidity of the Lewis acid (see Tables 1 and 2). Statistical analyses revealed that ZORA‐BP86/TZ2P performs equally as well as ZORA‐M06‐2X‐D3/QZ4P//ZORA‐BP86/TZ2P and significantly better than ZORA‐B3LYP‐D3(BJ)/QZ4P//ZORA‐BP86/TZ2P relative to the (TightPNO)DLPNO‐CCSD(T)/CBS(3,4/def2)//ZORA‐BP86/TZ2P data (see Table S4). Interestingly, only a poor linear correlation (*R* ^2^=0.62) is found when plotting the computed activation barriers vs. the corresponding HOMO_diene_–LUMO_ester_ gaps (see Figure S2 a). This result confirms that the HOMO–LUMO interactions, at variance with the current view, are not the main factor behind the computed reactivity trends.

To gain quantitative insight into the physical factors leading to the computed reactivity trend in the above‐mentioned LA‐catalyzed Diels–Alder reactions, we next turned to the activation strain model (ASM) of reactivity.^13^ This analysis involves decomposing the electronic energy (Δ*E*) into two terms: the strain (Δ*E*_strain_) that results from the distortion of the individual reactants and the interaction (Δ*E*_int_) between the deformed reactants along the reaction coordinate, defined in this case by the shorter of the two newly forming C⋅⋅⋅C bonds between isoprene and methyl acrylate. This critical reaction coordinate undergoes a well‐defined change throughout the reaction and has been used in the past in the analysis of similar reactions.^14^ Figure 1 a shows the corresponding activation strain diagrams (ASDs) from the reactant complexes to the transition states (see Figure S3 for the complete reaction profiles) for the uncatalyzed (none), TiCl_4_‐, and AlCl_3_‐catalyzed Diels–Alder reactions. The accelerated reactivity of the LA‐catalyzed reactions originates predominantly from a more stabilizing interaction energy along the entire reaction coordinate and also from a less destabilizing strain (albeit to a lesser extent). Specifically, the interaction energy becomes increasingly more stabilizing from LA=none(black)<TiCl_4_(red)<AlCl_3_(blue), and this is the same trend as the activation barriers. Thus, the reactivity trends are mainly caused by the trend in the interaction between the two reactants. Differences in the strain curves for the LA‐catalyzed reactions are similar along the reaction coordinate and are less destabilizing than for the uncatalyzed Diels–Alder reaction. Differences in Δ*E*_strain_ between the uncatalyzed and LA‐catalyzed Diels–Alder reaction can be ascribed to the higher asynchronicity of the latter which leads to a lower degree of deformation of the diene since one C−C bond forms ahead of the other C−C bond.^15^

**Figure 1:**
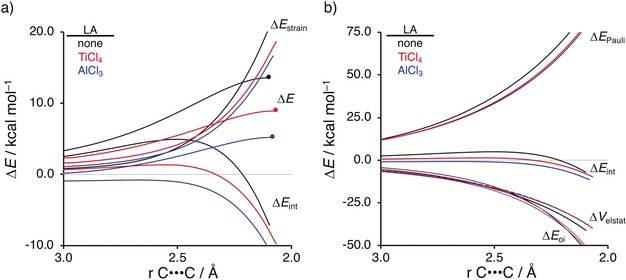
a) Activation strain analyses and b) energy decomposition analyses of the Diels–Alder reactions between isoprene and uncoordinated (none) as well as TiCl_4_‐ and AlCl_3_‐coordinated methyl acrylate complexes. Transition states are indicated by a dot. *r*(C⋅⋅⋅C) is the length of the shorter of the two C−C bonds forming between isoprene and methyl acrylate. Values computed at ZORA‐BP86/TZ2P.

The decisive role of the interaction energy on the observed reactivity trends prompted the analysis of the different contributors to the interaction energy using a canonical energy decomposition analysis (EDA).^10^ At this point, we note that concepts similar to those in our canonical EDA, in particular Pauli repulsion and orbital interaction (also referred to as relaxation or resonance), also feature, and have been successfully applied to reactions in other chemical‐bonding schemes such as DFT‐SAPT^16^ or valence bond (VB) theory.^17^ Our canonical EDA involves decomposing the Δ*E*_int_ between the reactants into three energy terms that are associated with the following physical factors: classical electrostatic interaction (Δ*V*_elstat_), Pauli repulsion (Δ*E*_Pauli_) between closed‐shell orbitals which is responsible for steric repulsion, and stabilizing orbital attractions (Δ*E*_oi_) that account, among others, for HOMO–LUMO interactions. The corresponding EDA results for the uncatalyzed (none), TiCl_4_‐, and AlCl_3_‐catalyzed Diels–Alder reactions are presented in Figure 1 b. Contrary to the commonly accepted view that LAs enhance the electrostatic and orbital interactions in catalyzed Diels–Alder reactions, we find that differences in the Δ*V*_elstat_ and Δ*E*_oi_ curves are minimal (the black, red, and blue curves are nearly superimposed). This clearly indicates that the Δ*E*_Pauli_ curves determine the differences in the Δ*E*_int_ curves and, thus, the activation barriers. The reduction of Δ*E*_Pauli_ for LA‐catalyzed Diels–Alder reactions is unprecedented and supports our previous findings involving LA‐catalyzed Michael addition reactions.^8^ We now, therefore, demonstrate the more general applicability of the reduction of Δ*E*_Pauli_ being the causal term behind the catalytic ability of LAs regardless of the type of reaction, that is, Michael addition or Diels–Alder reactions.^18^ Identical conclusions are found when explicit dispersion corrections are employed at both ZORA‐BP86‐D3(BJ)/TZ2P//ZORA‐BP86/TZ2P and ZORA‐M06‐2X‐D3/TZ2P//ZORA‐BP86/TZ2P (see Figures S6 and S7).

The origin of the less destabilizing Pauli repulsion for the LA‐catalyzed Diels–Alder reaction was investigated next by performing a Kohn–Sham molecular orbital (KS‐MO) analysis. The occupied molecular orbitals of the diene and ester, as well as TiCl_4_–ester and AlCl_3_–ester, were quantified at a geometry in which the shorter of the two C−C bonds forming between isoprene and methyl acrylate was kept at a fixed length (Figure 2 a). Performing this analysis at a consistent point along the reaction coordinate (near all transition structures), rather than the transition state alone, ensures that the results are not skewed by the position of the transition state.13c, 19

**Figure 2:**
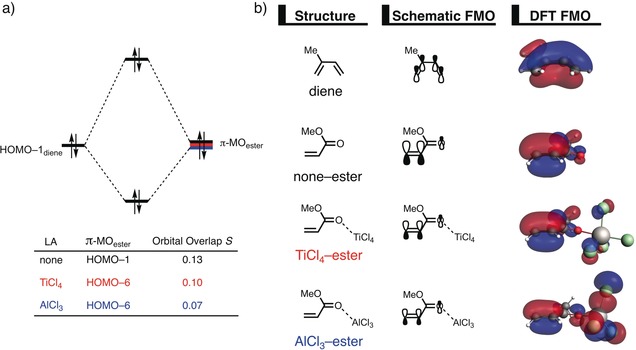
a) Molecular orbital diagram and the most significant occupied orbital overlaps of the Diels–Alder reactions between isoprene and uncoordinated (none) as well as TiCl_4_‐ or AlCl_3_‐coordinated methyl acrylate complexes. b) Key occupied orbitals (isovalue=0.03) computed at a geometry in which the shorter of the two C−C bonds forming between isoprene and methyl acrylate was kept at a fixed length of 2.097 Å at (ZORA‐BP86/TZ2P).

The occupied π‐MO_ester_ involved in this four‐electron‐two‐orbital interaction is the HOMO−1 for the uncatalyzed (none) reaction and the HOMO−6 for the TiCl_4_‐ and AlCl_3_‐catalyzed reactions. Importantly, the π‐MO_ester_ is the same π‐orbital located on the reactive C=C double bond in all three cases. The occupied MO of the diene responsible for the magnitude of the Pauli repulsion is the HOMO−1 where all carbon *p*‐orbitals are in‐phase.^20^ The orbital overlap between the π‐MO_ester_ and the HOMO−1_diene_ is the largest and most destabilizing for the uncatalyzed reaction (*S*=0.13), and smallest and least destabilizing for the AlCl_3_‐catalyzed reaction (*S*=0.07). The polarization of the π‐MO_ester_ of the catalyzed reaction away from the C=C double bond induced by the LA is the reason for the decreased ⟨HOMO−1_diene_|π‐MO_ester_⟩ overlap. Relatively strong donor–acceptor interactions between the σ* orbital of the LA and the π‐HOMO of methyl acrylate (see Table 1) result in charge transfer from methyl acrylate to the LA and manifest in a significantly smaller orbital amplitude on the C=C double bond (Figure 2 b), which is directly involved in the Diels–Alder reaction. Therefore, it can be concluded that the LA induces a significant reduction of the electron density at the reactive C=C double bond of the ester which results into a lower ⟨HOMO−1_diene_|π‐MO_ester_⟩ overlap and, ultimately, to a less destabilizing Pauli repulsion.

Lastly, we explored the counterintuitive finding from our EDA results that the strength of the orbital interactions is very similar for the uncatalyzed as well as TiCl_4_‐ and AlCl_3_‐catalyzed reactions, despite that the latter processes benefit from a more favorable HOMO_diene_–LUMO_ester_ gap (Figure S8). To this end, we applied the NOCV (natural orbitals for chemical valence)^21^ extension of the EDA method for the extreme situations represented by the uncatalyzed and AlCl_3_‐catalyzed Diels–Alder reactions. This approach identifies two main molecular orbital interactions that dominate the total orbital interactions in the herein studied transformations, namely, the normal electron demand (NED) HOMO_diene_–LUMO_ester_ and the inverse electron demand (IED) LUMO_diene_–π‐HOMO_ester_ interactions (*ρ*_1_ and *ρ*_2_, respectively, see Figure 3). As expected for an NED Diels–Alder reaction, the HOMO_diene_–LUMO_ester_ interaction is stronger than the LUMO_diene_–π‐HOMO_ester_ interaction in both cases (Δ*E*(*ρ*_1_)>Δ*E*(*ρ*_2_)). Not surprisingly, this primary NED interaction is significantly stronger in the LA‐catalyzed cycloaddition than in its uncatalyzed counterpart (ΔΔ*E*(*ρ*_1_)=5.8 kcal mol^−1^), which is consistent with the more favorable HOMO_diene_–LUMO_ester_ gap of 0.7 eV compared to 2.3 eV of the uncatalyzed reaction (see Figure S8 for the complete Kohn–Sham molecular‐orbital analysis). Despite that, the LA also weakens the IED LUMO_diene_–π‐HOMO_ester_ interaction to a nearly identical extent (ΔΔ*E*(*ρ*_2_)=−6.1 kcal mol^−1^). These combined interactions effectively offset and for this reason, the total orbital interactions between the deformed reactants are rather similar in both processes.

**Figure 3:**
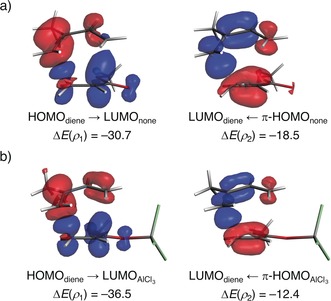
Contour plots of NOCV deformation densities Δ*ρ* and associated energies Δ*E*(*ρ*) (in kcal mol^−1^) for the normal electron demand (HOMO_diene_–LUMO_ester_) and inverse electron demand (LUMO_ester_–π‐HOMO_diene_) interactions for the a) uncatalyzed and b) AlCl_3_‐catalyzed Diels–Alder reactions between isoprene and methyl acrylate computed at a geometry in which the shorter of the two C−C bonds forming between isoprene and methyl acrylate was kept at a fixed length of 2.097 Å at ZORA‐BP86/TZ2P. Electron‐density charge flow: red→blue.

To conclude, our computational study, based on the activation strain model and canonical energy decomposition analysis, clearly reveals that LAs catalyze the Diels–Alder reaction between isoprene and methyl acrylate via an unexpected and unprecedented electronic mechanism: reduced four‐electron (Pauli) repulsion between the π‐systems of the dienophile and the incoming diene. The decrease in Pauli repulsion between the reactants stems from the concomitant polarization of the conjugated π‐system away from the C=C double bond when the LA binds to the carbonylic oxygen of methyl acrylate. To our surprise, coordination of a LA, although inducing a remarkable reduction of the HOMO_diene_–LUMO_ester_ gap, does not enhance the orbital interactions between both reactants. This is due to the fact that the LA does not only enhance the HOMO_diene_–LUMO_ester_ interaction but also weakens the LUMO_diene_–π‐HOMO_ester_ interaction to a nearly identical extent. Therefore, at variance with the current, well‐established view, HOMO–LUMO interactions should not be used to rationalize the reactivity trends, at least in LA‐catalyzed processes such as the Diels–Alder cycloaddition or Michael addition reactions.

## Conflict of interest

The authors declare no conflict of interest.

## Supporting information

As a service to our authors and readers, this journal provides supporting information supplied by the authors. Such materials are peer reviewed and may be re‐organized for online delivery, but are not copy‐edited or typeset. Technical support issues arising from supporting information (other than missing files) should be addressed to the authors.

Supplementary
