# Supplementary material for: How Lewis Acids Catalyze Diels–Alder Reactions
Source: Angew Chem Int Ed Engl. 2020 Feb 19;59(15):6201–6. doi: 10.1002/anie.201914582 (PMC7187354; doi:10.1002/anie.201914582)
Supplement: Supplementary file 1 — Supplementary [file ANIE-59-6201-s001.pdf]

Angewandte Chemie
Eine Zeitschrift der Gesellschaft Deutscher Chemiker

Supporting Information

How Lewis Acids Catalyze Diels–Alder Reactions

Pascal Vermeeren, Trevor A. Hamlin, Israel Fernández, and F. Matthias Bickelhaupt**

anie_201914582_sm_miscellaneous_information.pdf

Contents

Computational Details

Table S1: Energy decomposition analysis terms (in kcal mol^{-1}) computed on LA–ester complexes.

Table S2: Energy decomposition analysis terms (in kcal mol^{-1}) computed and LA···O=C distance (in Å) on LA–ester complexes, computed at ZORA-BP86-D3(BJ)/TZ2P.

Figure S1: Transition state structures for the uncatalyzed and Lewis acid-catalyzed Diels–Alder reaction between isoprene and methyl acrylate forming a 1,4-cycloadduct (left) or 1,3-cycloadduct (right), computed at ZORA-BP86/TZ2P.

Table S3: Electronic reaction barriers ($\Delta E^{\ddagger}$, in kcal mol^{-1}) computed for the uncatalyzed and LA-catalyzed Diels-Alder reaction between isoprene (diene) and methyl acrylate (ester) leading to the 1,4-cycloadduct computed at various DLPNO-CCSD(T)/basis-set//ZORA-BP86/TZ2P levels

Table S4: Statistical analysis of various XC functionals (kcal mol^{-1}): mean deviation $\Delta E^{\ddagger}_{\mathrm{MD}}$, mean absolute deviation $\Delta E^{\ddagger}_{\mathrm{MAD}}$, standard deviation mean deviation $\Delta E^{\ddagger}_{\mathrm{SD}}$, maximum negative $\Delta E^{\ddagger}_{\mathrm{max(-)}}$ and positive negative $\Delta E^{\ddagger}_{\mathrm{max(+)}}$ error relative to (TightPNO)DLPNO-CCSD(T)/ CBS(3,4/def2)//ZORA-BP86/TZ2P computed electronic reaction barriers of the uncatalyzed and catalyzed Diels-Alder reaction between isoprene (diene) and methyl acrylate (ester) leading to the 1,4-cycloadduct.

Figure S2: Activation barriers for the uncatalyzed and catalyzed Diels-Alder reactions versus the reactants' a) $\mathrm{HOMO_{diene}}$–$\mathrm{LUMO_{ester}}$ and b) $\mathrm{LUMO_{diene}}$–π-$\mathrm{MO_{ester}}$ gaps (Δε), computed at ZORA-BP86/TZ2P.

Figure S3: Complete a) activation strain analyses and b) energy decomposition analyses diagram from the reactants to the product of the Diels-Alder reactions between isoprene and none, $TiCl_4$, and $AlCl_3$ methyl acrylate complexes computed at ZORA-BP86/TZ2P.

Figure S4: Strain decomposition diagram of the Diels-Alder reactions between isoprene and none, $TiCl_4$, and $AlCl_3$ methyl acrylate complexes computed at ZORA-BP86/TZ2P.

Figure S5: a) Activation strain analyses and b) energy decomposition analyses of the uncatalyzed and LA-catalyzed Diels–Alder reactions between isoprene and LA–methyl acrylate complexes, computed at ZORA-BP86/TZ2P.

Figure S6: a) Activation strain analyses and b) energy decomposition analyses of the uncatalyzed and $AlCl_3$-catalyzed Diels–Alder reactions between isoprene and $AlCl_3$–methyl acrylate complexes, computed at ZORA-BP86-D3(BJ)/TZ42)//ZORA-BP86/TZ2P.

Figure S7: a) Activation strain analyses and b) energy decomposition analyses of the uncatalyzed and $AlCl_3$-catalyzed Diels–Alder reactions between isoprene and $AlCl_3$–methyl acrylate complexes, computed at ZORA-M06-2X-D3/TZ2P)//ZORA-BP86/TZ2P.

Figure S8: MO diagrams with calculated orbital energies and overlaps of the Diels-Alder reactions between diene and none, $TiCl_4$, and $AlCl_3$ for a) the normal electron demand $HOMO_{diene}$–$LUMO_{ester}$ interaction and b) inverse electron demand $LUMO_{diene}$–π-MO_{ester} interaction computed at consistent geometries with a shorter C•••C forming bond length between isoprene and methyl acrylate of 2.097Å at ZORA-BP86/TZ2P.

Table S5. Cartesian coordinates (in Å), energies (in kcal mol^{-1}), and number of imaginary frequencies of all stationary points, computed at ZORA-BP86/TZ2P.

Computational Details

All stationary points and vibrational analyses were carried out at ZORA-BP86/TZ2P[1,2 a,2b,3]using ADF.2018.104.[4] The BP86/TZ2P level has proven to be accurate in calculating the relative trends in activation and reaction energies for cycloadditions.[5] The zeroth-order regular approximation (ZORA) was used to account for scalar relativistic effects. This level is referred to as ZORA-BP86/TZ2P. The activation strain and energy decomposition analyses were performed using the PyFrag 2019 program.[6] The Domain Based Local Pair-Natural Coupled-Cluster (DLPNO-CCSD(T))[7] calculations, with both NormalPNO and TightPNO, were performed using Orca 4.0.1[8] using the def2-QZVPP[9] and CBS(3,4/def2)[10] basis set, repectively, on ZORA-BP86/TZ2P geometries. Additionally, ZORA-M06-2X-D3/QZ4P,[1, 11, 12 a,12b,3] ZORA-M06-2X-D3/TZ2P,[1,11,12,3] ZORA-B3LYP-D3(BJ)/QZ4P,[1,2,12,3] and ZORA-BP86-D3(BJ)/TZ2P[1,2,12,3] calculations were performed on ZORA-BP86/TZ2P geometries, in order to study the effect of meta hybrid and hybrid exchange-correlation functionals, as wel as dispersion corrections on the computed reactivity trends. Moreover, the NOCV (Natural Orbital for Chemical Valence) extension of the EDA method has also been used for further partitioning of the ΔE_{oi} term. The EDA-NOCV approach identifies the main molecular orbital interactions that dominate the total orbital interactions.[13]

---

[1] a) E. van Lenthe, E. J. Baerends, J. G. Snijders, *J. Chem. Phys.* **1993**, *99*, 4597; b) E. van Lenthe, E. J. Baerends, J. G. Snijders, *J. Chem. Phys.* **1994**, *101*, 9783.

[2] a) A. D. Becke, *Phys. Rev. A*, 1988, **38**, 3098; b) J. P. Perdew, *Phys. Rev. B: Condens. Matter Mater. Phys.* **1986**, *33*, 8822; c) A. D. Becke, *J. Chem. Phys.* **1993**, *98*, 1372.

[3] a) E. van Lenthe, E. J. Baerends, *J. Comput. Chem.* **2003**, *24*, 1142; b) M. Franchini, P. H. T. Philipsen, E. van Lenthe, L. Visscher, *J. Chem. Theory Comput.* **2014**, *10*, 1994.

[4] a) G. te Velde, F. M. Bickelhaupt, E. J. Baerends, C. Fonseca Guerra, S. J. A. van Gisbergen, J. G. Snijders, T. Ziegler, *J. Comput. Chem.* **2001**, *22*, 931; b) C. Fonseca Guerra, J. G. Snijders, G. te Velde, E. J. Baerends, *Theor. Chem. Acc.* **1998**, *99*, 391; c) ADF2018.104, SCM Theoretical Chemistry, Vrije Universiteit: Amsterdam (Netherlands). http://www.scm.com.

[5] a) T. A. Hamlin, D. Svatunek, S. Yu, L. Ridder, I. Infante, L. Visscher, F. M. Bickelhaupt, *Eur. J. Org. Chem.* **2019**, 378; b) A. Talbot, D. Devarajan, S. J. Gustafson, I. Fernández, F. M. Bickelhaupt, D. H. Ess, *J. Org. Chem.* **2015**, *80*, 548.

[6] X. Sun, T. M. Soini, J. Poater, T. A. Hamlin, F. M. Bickelhaupt, *J. Comp. Chem.*, **2019**, *40*, 2227.

[7] a) F. Neese, *WIREs Comput. Mol. Sci.* **2018**, *8*, e1327; b) C. Riplinger, B. Sandhoefer, A. Hansen, F. Neese, *J. Chem. Phys*. **2013**, *139*, 134101.

[8] F. Neese, *WIREs Comput. Mol. Sci*. **2018**, *8*, e1327.

[9] a) F. Weigend, R. Ahlrichs, *Phys. Chem. Chem. Phys.* **2005**, *7*, 3297; b) F. Weigend, *Phys. Chem. Chem. Phys.* **2006**, *8*, 1057.

[10] a) J. A. Montgomery, Jr., J. W. Ochterski, G. A. Petersson, *J. Chem. Phys.* **1994**, *101*, 5900; b) J. W. Ochterski, G. A. Petersson, and J. A. Montgomery, Jr., *J. Chem. Phys*. **1996**, *104*, 2598.

[11] Y. Zhao, D. G. Truhlar, *Theor. Chem. Acc.* **2008**, *120*, 215.

[12] a) S. Grimme, J. Antony, S. Ehrlich, H. Krieg, *J. Chem. Phys.* **2010**, *132*, 154104; b) S. Grimme, S. Ehrlich, L. Goerigk, *J. Comput. Chem.* **2011**, *32*, 1456; c) B. G. Johnson, P. M. W. Gill, J. A. Pople, *J. Chem. Phys.* **1993**, *98*, 5612; d) T.V. Russo, R.L. Martin, P. J. Hay, *J. Chem. Phys.* **1994**, *101*, 7729.

[13] M. P. Mitoraj, A. Michalak, T. A. Ziegler, *J. Chem. Theory Comput.* **2009**, *5*, 962.

Table S1: Energy decomposition analysis terms (in kcal mol^{-1}) computed on LA–ester complexes.[a]

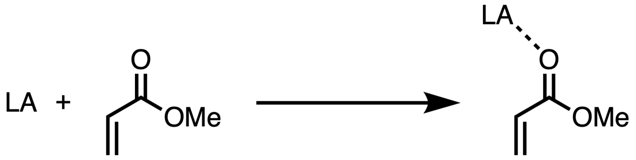

LA	ΔE	ΔE_{strain}	ΔE_{int}	ΔV_{elstat}	ΔE_{Pauli}	ΔE_{oi}
I_2	–5.2	0.4	–5.5	–13.9	18.6	–10.2
$SnCl_4$	–2.7	7.3	–10.0	–31.5	40.8	–19.2
$TiCl_4$	–3.6	10.9	–14.5	–41.5	50.6	–23.6
$ZnCl_2$	–10.4	6.8	–17.2	–41.5	46.8	–22.5
BF_3	–7.2	18.4	–25.6	–59.8	87.0	–52.8
$AlCl_3$	–26.9	10.6	–37.5	–65.7	74.4	–46.2

[a] The Lewis acid (LA) and methyl acrylate (ester) constitute the two interacting fragments. Computed at ZORA-BP86/TZ2P.

The trend in LA–ester complexation energy (ΔE) is determined by an interplay between the strain (ΔE_{strain}) and interaction energies (ΔE_{int}). For the analysis of the interaction energy see manuscript. The ΔE_{strain} is the largest for BF_3, which upon complexation is required to pyramidalize. This pyramidalization mode of deformation is also responsible for the relatively high destabilizing ΔE_{strain} of $AlCl_3$. Upon complexation $SnCl_4$ and $TiCl_4$, on the other hand, distort from tetrahedral to trigonal bipyramidal leading to a destabilizing ΔE_{strain} of ~10 kcal mol^{-1}. Furthermore, $ZnCl_2$ only bends from a linear geometry to a trigonal planar geometry and the I–I bond of I_2 slightly elongates upon complexation with the ester resulting in less destabilizing ΔE_{strain} of complexation.

Table S2: Energy decomposition analysis terms (in kcal mol^{-1}) computed and LA···O=C distance (in Å) on LA–ester complexes, computed at ZORA-BP86-D3(BJ)/TZ2P.

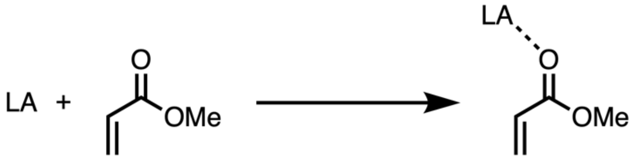

LA	ΔE_{int}	$\Delta V_{\mathrm{elstat}}$	$\Delta E_{\mathrm{Pauli}}$	ΔE_{oi}	ΔE_{disp}	r(LA···O=C)[a]
I_2	–9.1	–15.5	21.5	–11.5	–3.6	2.715 (2.745)
$SnCl_4$	–18.5	–39.1	52.6	–24.2	–7.8	2.412 (2.493)
$TiCl_4$	–22.7	–46.5	58.5	–26.4	–8.3	2.221 (2.250)
$ZnCl_2$	–21.8	–44.6	51.5	–23.9	–4.8	2.107 (2.129)
BF_3	–28.4	–58.1	88.4	–55.6	–3.1	1.728 (1.733)
$AlCl_3$	–44.0	–68.4	78.6	–47.4	–6.8	1.889 (1.898)

[a] The values in parenthesis were computed at the ZORA-BP86-TZ2P level (without D3(BJ) dispersion corrections).

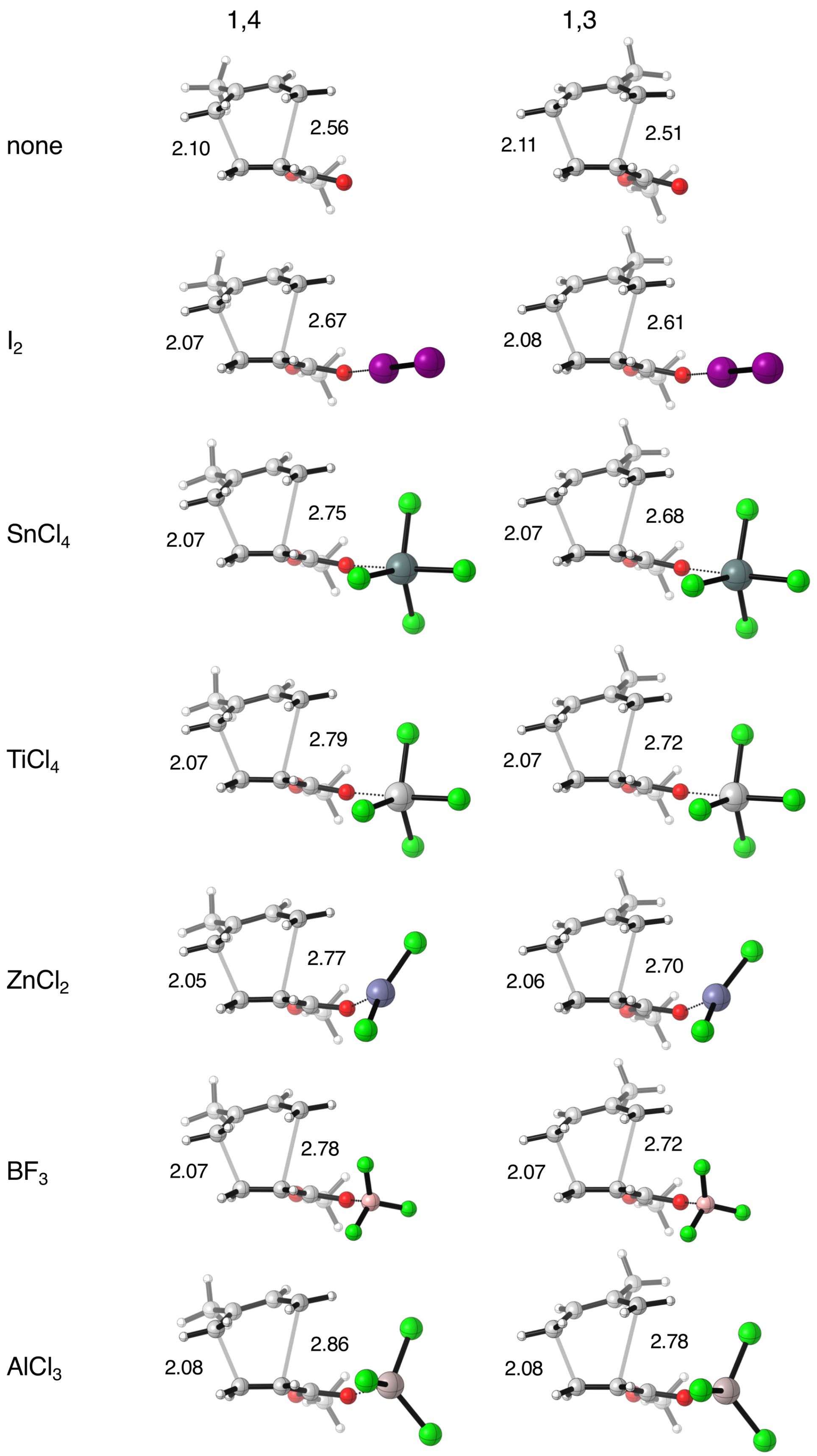

Figure S1: Transition state structures for the uncatalyzed and Lewis acid-catalyzed Diels–Alder reaction between isoprene and methyl acrylate forming a 1,4-cycloadduct (left) or 1,3-cycloadduct (right), computed at ZORA-BP86/TZ2P.

Table S3: Electronic reaction barriers ($\Delta E^{\ddagger}$, in kcal mol^{-1}) computed for the uncatalyzed and LA-catalyzed Diels-Alder reaction between isoprene (diene) and methyl acrylate (ester) leading to the 1,4-cycloadduct computed at various DLPNO-CCSD(T)/basis-set//ZORA-BP86/TZ2P levels.

LA	def2-TZVP (normalPNO)	def2-QZVPP (normalPNO)	CBS[a] (tightPNO)
none	16.7	16.5	16.2
I_2	14.3	14.6	15.0
$SnCl_4$	13.7	13.5	12.3
$TiCl_4$	12.2	12.8	11.8
$ZnCl_2$	11.4	11.4	10.8
BF_3	10.7	10.6	10.0
$AlCl_3$	9.0	8.1	7.6

[a] CBS(3/4,def2)

Table S4: Statistical analysis of the used XC functionals (in kcal mol^{-1}): mean deviation $\Delta E^{\ddagger}_{MD}$, mean absolute deviation $\Delta E^{\ddagger}_{MAD}$, standard deviation mean deviation $\Delta E^{\ddagger}_{SD}$, maximum negative $\Delta E^{\ddagger}_{max(-)}$ and positive negative $\Delta E^{\ddagger}_{max(+)}$ error relative to (TightPNO)DLPNO-CCSD(T)/ CBS(3,4/def2)//ZORA-BP86/TZ2P computed electronic reaction barriers of the uncatalyzed and LA-catalyzed Diels-Alder reaction between isoprene (diene) and methyl acrylate (ester) leading to the 1,4-cycloadduct.

	ZORA-BP86/TZ2P	ZORA-M06-2X-D3/QZ4P// ZORA-BP86/TZ2P	ZORA-B3LYP-D3(BJ)/QZ4P// ZORA-BP86/TZ2P
$\Delta E^{\ddagger}_{MD}$	2.6	2.2	5.4
$\Delta E^{\ddagger}_{MAD}$	2.6	2.2	2.2
$\Delta E^{\ddagger}_{SD}$	0.7	0.6	0.7
$\Delta E^{\ddagger}_{max(-)}$	2.9	2.9	6.2
$\Delta E^{\ddagger}_{max(+)}$	[a]	[a]	[a]

[a] No computed reaction barrier higher than the (TightPNO)DLPNO-CCSD(T)/ CBS(3,4/def2)//ZORA-BP86/TZ2P value.

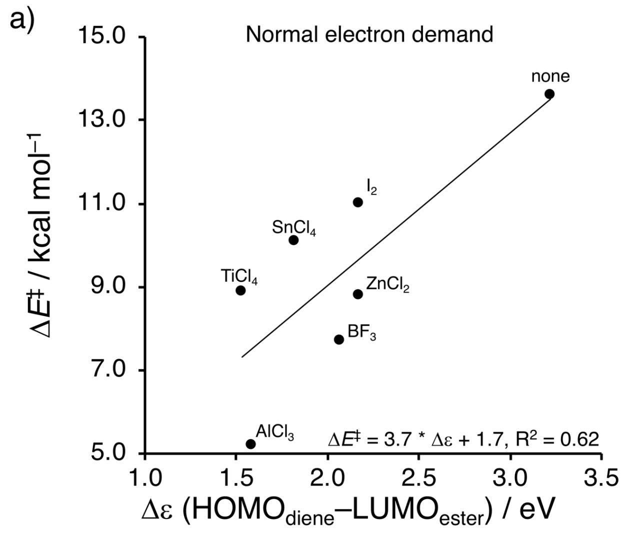

Figure S2: Activation barriers for the uncatalyzed and LA-catalyzed Diels-Alder reactions versus the reactants' a) $HOMO_{diene}$–$LUMO_{ester}$ and b) $LUMO_{diene}$–π-MO_{ester} gaps (Δε), computed at ZORA-BP86/TZ2P.

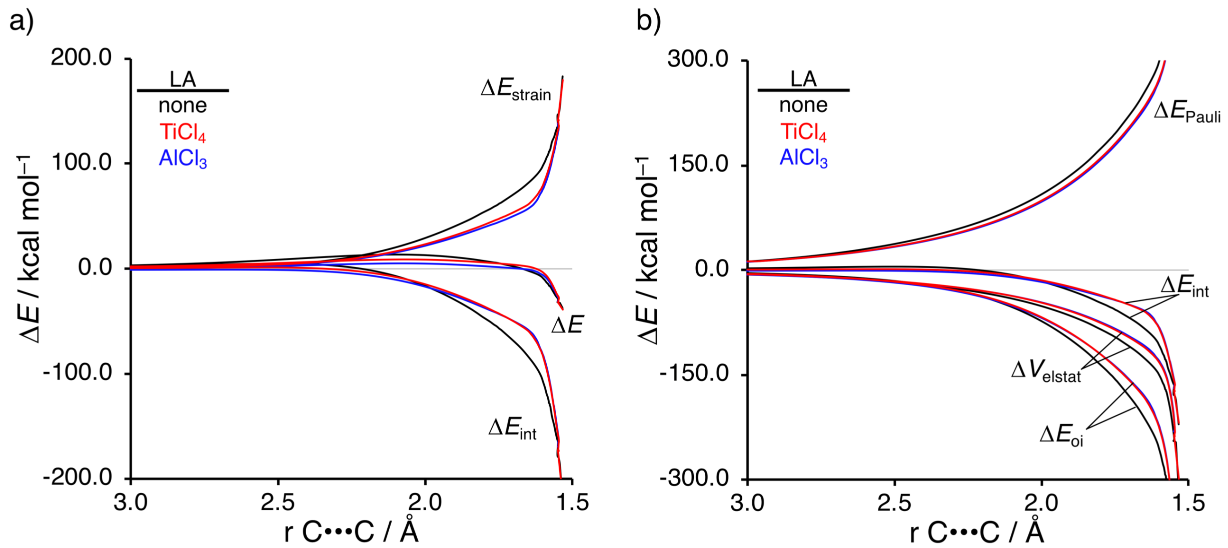

Figure S3: Complete a) activation strain analyses and b) energy decomposition analyses diagram from the reactants to the product of the Diels-Alder reactions between isoprene and none, $TiCl_4$, and $AlCl_3$ methyl acrylate complexes computed at ZORA-BP86/TZ2P.

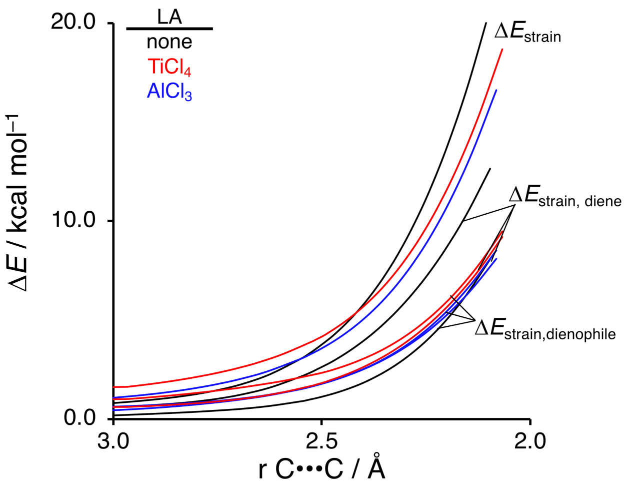

Figure S4: Strain decomposition diagram of the Diels-Alder reactions between isoprene and none, $TiCl_4$, and $AlCl_3$ methyl acrylate complexes computed at ZORA-BP86/TZ2P.

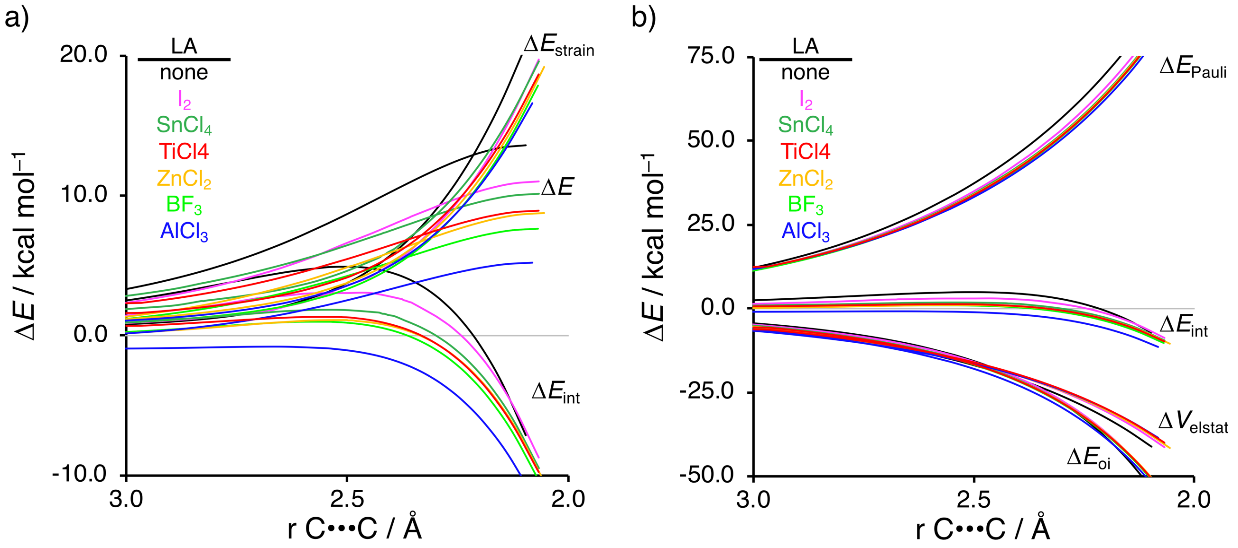

Figure S5: a) Activation strain analyses and b) energy decomposition analyses of the uncatalyzed and LA-catalyzed Diels–Alder reactions between isoprene and LA–methyl acrylate complexes, computed at ZORA-BP86/TZ2P.

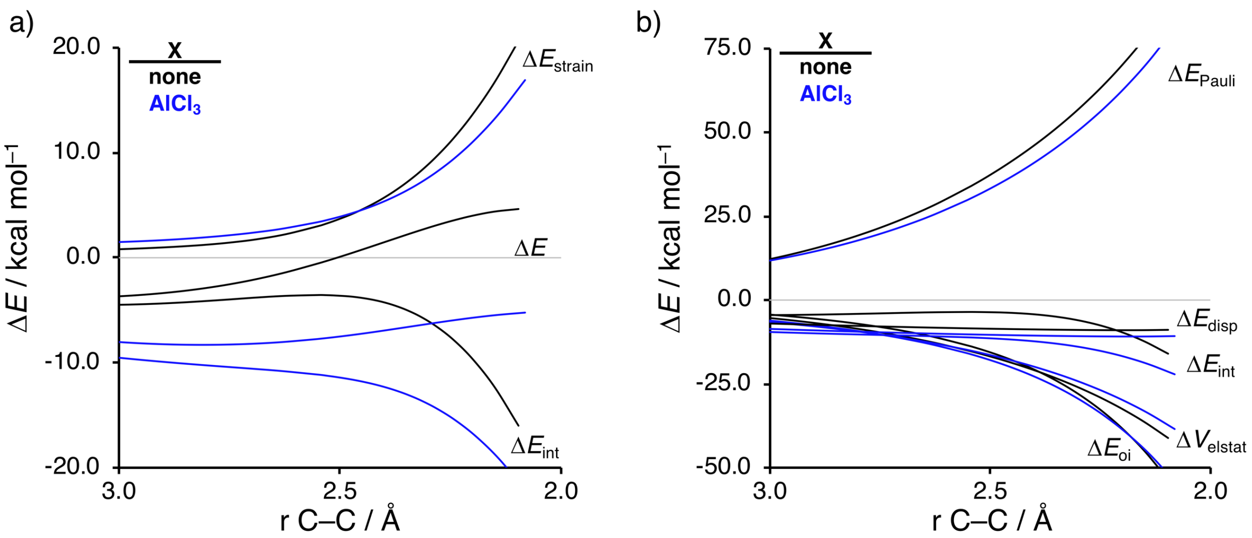

Figure S6: a) Activation strain analyses and b) energy decomposition analyses of the uncatalyzed and $AlCl_3$-catalyzed Diels–Alder reactions between isoprene and $AlCl_3$–methyl acrylate complexes, computed at ZORA-BP86-D3(BJ)/TZ2P//ZORA-BP86/TZ2P.

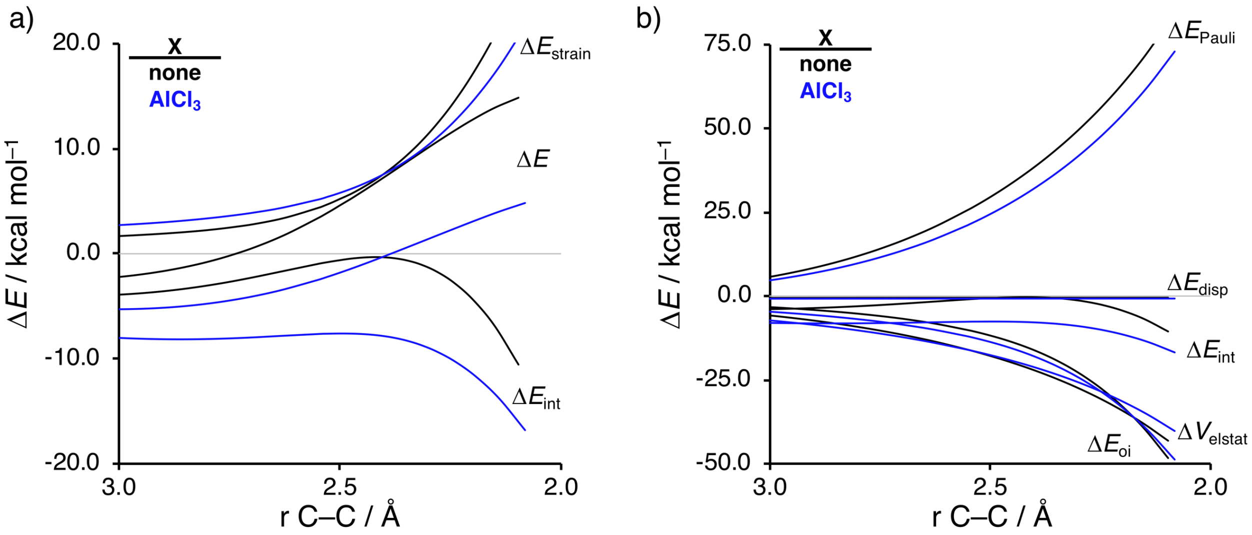

Figure S7: a) Activation strain analyses and b) energy decomposition analyses of the uncatalyzed and $AlCl_3$-catalyzed Diels–Alder reactions between isoprene and $AlCl_3$–methyl acrylate complexes, computed at ZORA-M06-2X-D3/TZ2P//ZORA-BP86/TZ2P.

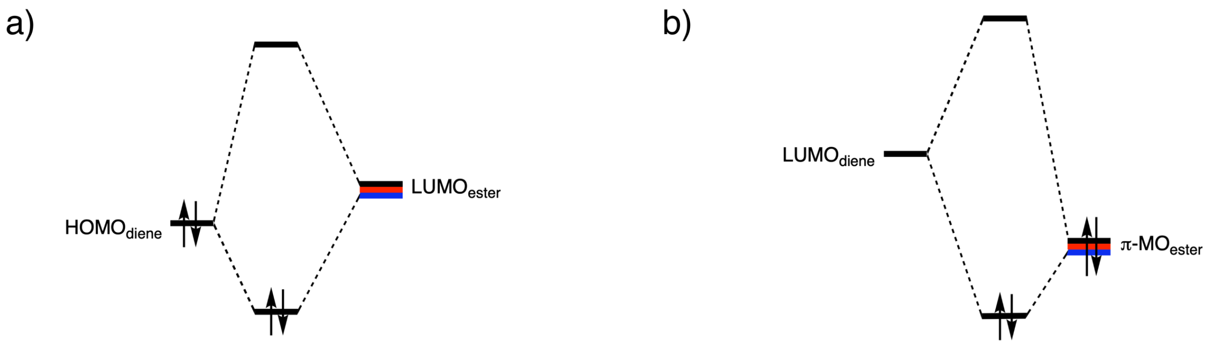

a)

X	Δε HOMO$_{diene}$–LUMO$_{ester}$ (eV)	Orbital Overlap *S*
none	2.3	0.20
$TiCl_4$	0.9	0.13
$AlCl_3$	0.7	0.17

b)

X	Δε LUMO$_{diene}$–π-MOester (eV)		Orbital Overlap *S*
none	HOMO–1	4.6	0.17
	HOMO–2	5.2	0.04
$TiCl_4$	HOMO–5	5.6	0.13
	HOMO–6	5.8	0.07
$AlCl_3$	HOMO–6	6.1	0.09
	HOMO–7	6.1	0.09
	HOMO–8	6.2	0.07

Figure S8: MO diagrams with calculated orbital energies and overlaps of the Diels-Alder reactions between isoprene and none, $TiCl_4$, and $AlCl_3$ methyl acrylate complexes for a) the normal electron demand HOMO$_{diene}$–LUMO$_{ester}$ interaction and b) inverse electron demand LUMO$_{diene}$–π-MO$_{ester}$ interaction computed at consistent geometries with a shorter C•••C forming bond length between isoprene and methyl acrylate of 2.097Å at ZORA-BP86/TZ2P.

The orbital gaps and overlaps of the normal and inverse electron demand interaction at consistent geometries are provided in Figure S6. Coordination of a Lewis acid strengthens the normal electron demand (NED) interaction and simultaneously weakens the inverse electron demand (IED) interaction, the effects of which effectively cancel. Thus, coordination of a Lewis acid does not significantly modulate the strength of the orbital interactions between isoprene (diene) and methyl acrylate (ester). The energy gap for the NED, HOMO$_{diene}$–LUMO$_{ester}$, interaction decrease and ranges from 2.3 eV for the uncatalyzed to 0.7 eV for the $AlCl_3$-catalyzed reaction (Figure S6a). This reduction in orbital energy gap is large enough to overcome the slight decrease of the orbital overlap and, therefore, coordination of a Lewis acid leads to a stronger HOMO$_{diene}$–LUMO$_{ester}$ NED interaction. The IED interaction is also modulated by coordination of a Lewis acid. Specifically, the Lewis acid stabilize all molecular orbitals of the ester and lead to an increased LUMO$_{diene}$–π-MO$_{ester}$ inverse electron orbital energy gap (see Figure S6b). This, together with a reduced orbital overlap, results in a weaker inverse electron demand orbital interaction compared to the uncatalyzed reaction.

Table S5: Cartesian coordinates (in Å), energies (in kcal mol^{-1}), and number of imaginary frequencies of all stationary points, computed at ZORA-BP86/TZ2P.

isoprene (diene)

E = −1672.09
H = −1598.97
G **=** **−**1621.55
N_{imag} = 0

C	-0.807690	0.084124	1.187135
C	-1.815891	0.348117	0.143317
C	-2.376003	-0.636449	-0.580083
C	-0.775888	-0.977510	2.003822
H	-0.043147	0.859137	1.310706
H	-3.130693	-0.423018	-1.337041
H	-2.083625	-1.677583	-0.447077
H	-1.544909	-1.750154	1.967192
H	0.015457	-1.098299	2.742624
H	-2.894182	1.920568	-0.887299
C	-2.156746	1.800544	-0.084972
H	-2.558638	2.260516	0.830324
H	-1.255647	2.372544	-0.355269

I_2

E = −57.68
H = −55.07
G **=** **−**73.65
N_{imag} = 0

I	-0.317347	-0.299315	0.000155
I	-2.977951	0.138920	-0.000120

$SnCl_4$

E = −351.95
H = −343.59
G **=** **−**317.56
N_{imag} = 0

Sn	-0.711223	1.415023	-0.000001
Cl	1.193004	2.743102	0.000001
Cl	-1.972288	1.870609	-1.895356
Cl	-1.972285	1.870608	1.895355
Cl	-0.094994	-0.823294	0.000001

$TiCl_4$

E = −497.98
H = −489.28
G **=** −514.53
N_{imag} = 0

Ti	-0.000000	0.000000	-0.000000
Cl	-1.259686	1.259686	1.259687
Cl	1.259686	1.259686	-1.259686
Cl	-1.259686	-1.259686	-1.259686
Cl	1.259686	-1.259686	1.259686

$ZnCl_2$
E = −161.45
H = −156.57
***G* =** −176.21
N_{imag} **=** 0

Zn	0.000000	0.000000	-0.000000
Cl	-0.000000	0.000000	-2.073818
Cl	0.000000	0.000000	2.073818

BF_3
E = −534.18
H = −523.88
***G* =** −542.09
N_{imag} **=** 0

B	0.000013	-0.000000	0.000000
F	1.324148	0.000000	0.000000
F	-0.662081	-1.146729	0.000000
F	-0.662081	1.146729	0.000000

$AlCl_3$
E = −328.3
H = −321.39
***G* =** −343.77
N_{imag} **=** 0

Al	0.000083	-0.000000	0.000000
Cl	1.040157	-1.801893	-0.000000
Cl	1.040157	1.801893	-0.000000
Cl	-2.080397	0.000000	0.000000

methyl acrylate (ester)
E = −1617.96
H = −1555.32
***G* =** −1579.49
N_{imag} **=** 0

O	1.274218	0.801086	-0.615814
C	2.122912	1.848410	-0.095924
H	-0.570034	-0.274783	-1.916648
H	-0.932075	-2.103538	-1.944866
H	0.954568	-2.503607	-0.403883
H	3.164775	1.681966	-0.394906
H	2.067525	1.879173	0.998875
H	1.739083	2.775751	-0.529253
O	2.517681	-0.690730	0.570119
C	-0.320233	-1.279547	-1.578863
C	0.700524	-1.499402	-0.744725
C	1.593624	-0.453499	-0.189039

I_2–ester
E = −1680.82
H = −1614.46
G **=** −1649.33
$\boldsymbol{N_{imag}}$ **=** 0

C	4.062835	2.477686	-0.000007
C	3.095856	1.554721	-0.000029
C	3.328784	0.096287	-0.000104
O	2.430140	-0.745298	-0.000125
O	4.632368	-0.251354	-0.000163
C	4.897162	-1.676495	-0.000244
H	5.115946	2.200151	-0.000063
H	3.819326	3.539390	0.000045
H	2.039972	1.828272	0.000007
H	4.467001	-2.142534	-0.893984
H	4.467005	-2.142637	0.893445
H	5.986107	-1.764735	-0.000252
I	-0.273747	-0.273012	0.000047
I	-2.974234	0.164377	0.000228

$SnCl_4$–ester
E = −1972.68
H = −1900.28
G = −1942.06
$\boldsymbol{N_{imag}}$ **=** 0

C	-3.845165	-2.069953	-0.000082
C	-2.657754	-1.454668	-0.000040
C	-2.486764	0.008724	0.000029
O	-1.393488	0.590026	0.000056
O	-3.633936	0.700411	0.000069
C	-3.505015	2.147506	0.000141
Sn	1.031954	0.012721	-0.000009
Cl	0.421765	-1.051798	-2.002734
Cl	0.421823	-1.052027	2.002613
Cl	1.104572	2.348151	0.000123
H	-4.779123	-1.510194	-0.000058
H	-3.906809	-3.157237	-0.000138
H	-1.725366	-2.016343	-0.000056
H	-2.965172	2.477409	-0.894008
H	-2.965154	2.477320	0.894312
H	-4.530888	2.522136	0.000169
Cl	3.343106	-0.471678	-0.000069

$TiCl_4$–ester
E = −2119.51
H = −204678
G **=** −2086.83
$\boldsymbol{N_{imag}}$ **=** 0

C	-3.637184	-2.010699	-0.000199
C	-2.421949	-1.451441	-0.000128
C	-2.185390	0.000947	-0.000025

O	-1.063066	0.525905	0.000042
O	-3.292111	0.750374	-0.000011
C	-3.089250	2.190033	0.000088
Ti	1.134267	0.033095	0.000091
Cl	0.643692	-0.973481	-1.928901
Cl	0.643553	-0.973758	1.928903
Cl	1.353825	2.238620	0.000264
H	-4.544184	-1.408160	-0.000181
H	-3.749306	-3.093921	-0.000278
H	-1.514872	-2.053155	-0.000144
H	-2.533329	2.490871	-0.894337
H	-2.533385	2.490755	0.894587
H	-4.094742	2.616064	0.000084
Cl	3.289279	-0.470450	0.000132

$ZnCl_2$-ester

E = −1789.78

H = −1721.75

***G* =** 1755.09

N_{imag} = 0

C	3.352334	1.694607	-0.442070
C	2.116697	1.237062	-0.209964
C	1.797628	-0.179591	0.028587
O	0.645384	-0.622731	0.165727
O	2.855204	-0.994298	0.091670
C	2.567735	-2.403072	0.310421
Zn	-1.336773	0.141799	0.027480
Cl	-2.457167	-1.562640	-0.549313
H	4.215331	1.030635	-0.466536
H	3.525929	2.755725	-0.615657
H	1.248350	1.897663	-0.178908
H	1.924236	-2.784986	-0.489179
H	2.069495	-2.538447	1.276448
H	3.543119	-2.893954	0.300826
Cl	-1.292930	2.217204	0.517729

BF_3-ester

E = −2159.31

H = −2085.18

***G* =** −2116.88

N_{imag} = 0

H	-1.564865	-1.738414	-2.076116
H	0.678842	-1.457271	-1.084680
H	1.054405	3.263932	-0.836112
H	0.385145	2.892568	0.781913
H	-0.664670	3.667726	-0.464446
B	2.840079	-0.394206	0.054776
F	3.830884	0.331408	0.633999
F	2.220561	-1.314148	0.863812
F	3.087613	-0.833322	-1.222076
C	-1.231287	-0.766697	-1.715286

C	-0.013567	-0.623595	-1.179439
C	0.498034	0.656776	-0.674170
O	1.627875	0.824236	-0.168422
O	-0.332524	1.689215	-0.781163
C	0.154784	2.968738	-0.285932
H	-1.921030	0.070809	-1.807891

$AlCl_3$-ester

E = -1973.18

H = -1902.58

***G* =** -1937.08

N_{imag} = 0

Cl	-1.322224	1.327005	1.805576
H	-0.251070	-2.892980	-1.865932
H	0.318380	-3.078069	-0.176969
Cl	-3.646645	-1.056732	0.429799
H	1.401975	-3.542092	-1.543796
H	3.019813	0.176273	-1.675951
H	2.802393	2.007169	-1.403587
H	0.476949	1.637160	-0.665468
C	2.380691	1.003897	-1.371888
C	1.118301	0.810817	-0.970398
C	0.487412	-0.509361	-0.902787
O	-0.693222	-0.702737	-0.525991
O	1.235775	-1.535668	-1.267887
C	0.621394	-2.858357	-1.205674
Al	-2.131906	0.378648	0.078110
Cl	-2.456854	1.698828	-1.562255

TS: 1,4_none-ester-diene

E = -3276.44

H = -3139.83

***G* =** -3173.48

N_{imag} = 1,-398.204i cm^{-1}

C	-1.168455	0.166132	1.424554
C	-2.024411	0.365414	0.309880
C	-2.270294	-0.691900	-0.562709
C	-0.562186	-1.027317	1.714001
H	-0.848167	1.056262	1.972524
H	-2.907421	-0.520635	-1.431413
H	-2.307345	-1.707603	-0.173684
H	-0.949278	-1.974573	1.349259
H	0.200761	-1.093174	2.487881
C	-0.425316	-1.089660	-1.474486
C	0.574622	-1.392960	-0.551767
C	1.654496	-0.477793	-0.174652
O	2.679065	-0.799061	0.411116
O	1.413545	0.815837	-0.583936
C	2.462729	1.746819	-0.254927
H	-0.360480	-0.146073	-2.012010
H	-0.863407	-1.914656	-2.035853

H	0.765872	-2.424879	-0.262968
H	3.408386	1.447769	-0.723659
H	2.611347	1.798605	0.831084
H	2.130760	2.713936	-0.643969
C	-2.408293	1.775338	-0.062528
H	-2.714115	2.343869	0.826443
H	-1.554366	2.310967	-0.506979
H	-3.233543	1.797032	-0.784194

TS: 1,3_none-ester-diene
E = −3275.84
H = −3139.83
G **=** −3173.48
N_{imag} **=** 1,−406.525i cm^{-1}

C	-0.078647	-0.245056	1.659945
C	-1.435352	0.003202	1.333315
C	-2.189459	-0.846090	0.535400
C	0.535258	-1.393429	1.211181
C	0.718565	0.844537	2.339761
H	-3.208161	-0.557923	0.275304
H	-2.028254	-1.921727	0.589028
H	-0.034269	-2.281371	0.953887
H	1.609399	-1.534909	1.325326
C	-1.245637	-0.819181	-1.351133
C	0.105202	-1.147303	-1.253498
C	1.197475	-0.175750	-1.385486
O	2.369640	-0.457474	-1.589254
O	0.755178	1.123816	-1.289069
C	1.787261	2.116776	-1.449680
H	-1.511678	0.206672	-1.593567
H	-1.929745	-1.580643	-1.724620
H	0.434093	-2.177830	-1.373212
H	2.260241	2.027497	-2.435381
H	2.559856	2.004725	-0.678816
H	1.283072	3.082622	-1.351736
H	-1.823749	1.005209	1.533455
H	0.429752	0.928563	3.397996
H	0.542087	1.823865	1.874709
H	1.793941	0.633033	2.306668

P: 1,4_none-ester-diene
E = −3327.52
H = −3187.93
G **=** −3220.06
N_{imag} **=** 0

C	1.400587	-1.657088	-0.604180
C	2.468070	-0.921499	-0.259373
C	2.366605	0.173196	0.779165
C	0.010815	-1.473168	-0.053560
H	1.523251	-2.445908	-1.351692
H	2.430344	1.156651	0.278947

H	3.247101	0.130548	1.441077
H	-0.247944	-2.324612	0.599327
H	-0.713841	-1.489886	-0.880067
C	1.088012	0.086086	1.619769
C	-0.151473	-0.166888	0.738443
C	-0.483229	1.005338	-0.186676
O	-0.805350	0.926787	-1.355092
O	-0.428967	2.191149	0.487859
C	-0.794327	3.351271	-0.293789
H	0.955759	0.994430	2.219475
H	1.173114	-0.758466	2.320283
H	-1.030495	-0.251805	1.399552
H	-1.826886	3.262701	-0.652459
H	-0.126792	3.459009	-1.156932
H	-0.691948	4.203186	0.383778
C	3.820544	-1.120441	-0.885649
H	4.172224	-0.193625	-1.367113
H	4.575551	-1.383274	-0.127390
H	3.804068	-1.912574	-1.644659

P: 1,3_none-ester-diene
E = −3327.62
H = −3188.03
***G* =** −3220.11
N_{imag} **=** 0

C	2.678607	-1.990660	-0.380557
C	3.927733	-1.564446	-0.140359
C	4.268741	-0.396795	0.746683
C	1.467782	-1.302984	0.212042
H	4.763297	-2.084234	-0.617831
H	4.600500	0.457792	0.129323
H	5.135414	-0.649274	1.377846
H	1.020425	-1.956989	0.980501
H	0.704166	-1.185982	-0.571085
C	3.090719	0.020209	1.634437
C	1.774166	0.067880	0.832093
C	1.761235	1.169058	-0.228970
O	1.400102	1.049342	-1.382605
O	2.169506	2.359441	0.299584
C	2.135038	3.475601	-0.618739
H	3.284625	0.987844	2.112214
H	2.961788	-0.722037	2.436687
H	0.960697	0.340933	1.525170
H	1.112745	3.646262	-0.977162
H	2.786169	3.284803	-1.480056
H	2.492672	4.335387	-0.045881
C	2.386821	-3.174514	-1.260126
H	1.800699	-3.935424	-0.719746
H	1.783711	-2.875422	-2.131731
H	3.308058	-3.647128	-1.623119

TS: 1,4_I$_2$-ester-diene

E = −3341.88
H = −3202.08
***G* =** −3243.87
***N*$_{imag}$ =** 1,−373.992i cm^{-1}

C	3.481736	-1.235300	-1.425924
C	4.528874	-1.584758	-0.527614
C	4.220908	-2.133766	0.715580
C	2.154358	-1.421955	-1.164201
H	3.758980	-0.653630	-2.309158
H	5.039323	-2.397946	1.386337
H	3.339577	-2.764641	0.814735
H	1.801901	-2.108917	-0.400026
H	1.388938	-1.013969	-1.822219
C	3.322228	-0.608250	1.781927
C	2.153202	-0.142952	1.179966
C	2.061521	1.104621	0.449925
O	1.012708	1.662078	0.083212
O	3.266943	1.692165	0.209026
C	3.210595	2.954167	-0.493195
H	4.185186	0.053829	1.812700
H	3.210861	-1.259729	2.648386
H	1.190583	-0.600190	1.402457
H	2.636151	3.689537	0.081969
H	2.744218	2.828226	-1.477559
H	4.251239	3.273281	-0.596045
C	5.925979	-1.114332	-0.837073
H	5.998336	-0.017574	-0.767862
H	6.664781	-1.548359	-0.153596
H	6.206880	-1.385793	-1.864465
I	-1.388463	0.607969	0.279304
I	-3.925495	-0.465017	0.410630

TS: 1,3_I$_2$-ester-diene

E = −3341.05
H = −3201.20
***G* =** −3240.52
***N*$_{imag}$ =** 1,−384.293i cm^{-1}

C	-3.533936	-1.323975	1.294273
C	-4.534128	-1.743624	0.377933
C	-4.250502	-2.281450	-0.870767
C	-2.204846	-1.480411	0.981509
H	-5.562460	-1.435995	0.585317
H	-5.080715	-2.546673	-1.525479
H	-3.363688	-2.900371	-0.999860
H	-1.868960	-2.192749	0.234251
H	-1.424033	-1.044323	1.603521
C	-3.367198	-0.743526	-1.953058
C	-2.232021	-0.244210	-1.315692
C	-2.196984	1.025998	-0.613100
O	-1.175688	1.622226	-0.234032

O	-3.425184	1.579811	-0.421995
C	-3.429178	2.866130	0.238189
H	-4.247556	-0.108060	-2.013487
H	-3.213713	-1.405108	-2.805106
H	-1.249038	-0.669766	-1.509386
H	-2.863979	3.599844	-0.348023
H	-2.985147	2.789399	1.237600
H	-4.482229	3.152483	0.303016
C	-3.957493	-0.565670	2.531392
H	-4.468608	-1.237904	3.235973
H	-3.094618	-0.134102	3.051788
H	-4.659493	0.243562	2.288063
I	1.275277	0.653809	-0.368208
I	3.851780	-0.320535	-0.444758

P: 1,4_I_2-ester-diene
E = −3389.57
H = −3247.39
***G* =** −3285.68
N_{imag} **=** 0

C	3.420944	-1.804465	-0.721925
C	4.643654	-1.416845	-0.327882
C	4.847210	-0.549195	0.891650
C	2.135456	-1.427319	-0.036282
H	3.327724	-2.447140	-1.601725
H	5.222700	0.436023	0.569610
H	5.653803	-0.980113	1.507569
H	1.747278	-2.285010	0.538570
H	1.357797	-1.200819	-0.779279
C	3.586660	-0.384842	1.753837
C	2.300759	-0.228042	0.926622
C	2.147839	1.085594	0.174597
O	1.175177	1.380535	-0.515464
O	3.167159	1.947898	0.351723
C	3.026761	3.230040	-0.314877
H	3.707935	0.456625	2.448096
H	3.454636	-1.286983	2.369958
H	1.432132	-0.230016	1.606596
H	2.121473	3.740242	0.032020
H	2.968877	3.087328	-1.399575
H	3.922473	3.793294	-0.042441
C	5.889130	-1.808652	-1.074097
H	6.450449	-0.918597	-1.400785
H	6.570733	-2.387047	-0.430352
H	5.658845	-2.411950	-1.960942
I	-1.372755	0.396785	-0.296513
I	-3.953729	-0.464952	-0.011644

P: 1,3_I_2-ester-diene
E = −3389.55
H = −3246.77
***G* =** −3287.01
***N*imag =** 0

C	-3.483548	-1.906738	0.412325
C	-4.706197	-1.440380	0.115337
C	-4.993231	-0.390074	-0.922120
C	-2.240924	-1.386215	-0.274823
H	-5.567549	-1.843248	0.656042
H	-5.360159	0.524863	-0.428870
H	-5.822767	-0.727297	-1.564145
H	-1.884598	-2.132678	-1.004620
H	-1.424456	-1.282203	0.454831
C	-3.773702	-0.068302	-1.798959
C	-2.456963	-0.042067	-1.006375
C	-2.285535	1.123712	-0.042555
O	-1.307990	1.287972	0.683267
O	-3.294518	2.014978	-0.069998
C	-3.138251	3.166755	0.799878
H	-3.924937	0.877536	-2.334500
H	-3.664750	-0.852740	-2.562923
H	-1.615895	0.088495	-1.708672
H	-2.226691	3.716412	0.540770
H	-3.082445	2.845191	1.845705
H	-4.027011	3.778158	0.626562
C	-3.256324	-2.987847	1.432793
H	-2.736560	-3.850113	0.985162
H	-2.614359	-2.630266	2.253020
H	-4.200994	-3.344165	1.861941
I	1.252939	0.400439	0.258785
I	3.839409	-0.354145	-0.212482

TS: 1,4_$SnCl_4$-ester-diene
E = −3634.63
H = −3488.75
***G* =** −3537.27
***N*imag =** 1,−347.189i cm^{-1}

C	-3.338832	-0.684549	1.539429
C	-4.506070	-0.625368	0.721321
C	-4.559412	-1.336938	-0.474382
C	-2.233568	-1.427472	1.249942
H	-5.472613	-1.288592	-1.068385
H	-4.014234	-2.274718	-0.562292
H	-2.245291	-2.227315	0.514245
H	-1.322611	-1.332600	1.838930
C	-3.243332	-0.370881	-1.741653
C	-1.925186	-0.405105	-1.285949
C	-1.268168	0.720623	-0.678335
O	-0.040006	0.837922	-0.447759
O	-2.083118	1.759913	-0.387480

C	-1.447688	2.925202	0.193602
Sn	2.047437	-0.176511	-0.362780
Cl	1.257487	-1.820338	-1.862476
Cl	1.445603	-0.361223	1.913530
Cl	2.653506	1.908214	-1.256257
H	-3.759764	0.586314	-1.765609
H	-3.506802	-1.055904	-2.547042
H	-1.268320	-1.235142	-1.533674
H	-0.682501	3.321227	-0.482835
H	-0.984067	2.669505	1.153154
H	-2.255110	3.648534	0.332745
H	-3.287675	0.012945	2.379649
C	-5.570340	0.381118	1.066890
H	-5.153178	1.395985	1.144004
H	-6.379066	0.392134	0.327310
H	-6.011791	0.146549	2.047284
Cl	4.242812	-1.057007	-0.125346

TS: 1,3_$SnCl_4$-ester-diene

E = −3633.62

H = −3487.69

***G* =** −3535.61

N_{imag} **=** 1,−365.260i cm^{-1}

C	-3.286507	-0.733629	1.523503
C	-4.439998	-0.704291	0.691554
C	-4.534098	-1.370738	-0.522408
C	-2.192813	-1.478679	1.161417
H	-5.454860	-1.276863	-1.097985
H	-4.014771	-2.318425	-0.657293
H	-2.255937	-2.275772	0.426591
H	-1.255413	-1.408339	1.712218
C	-3.200767	-0.408951	-1.785044
C	-1.895473	-0.442207	-1.294923
C	-1.251777	0.696488	-0.688040
O	-0.028500	0.825291	-0.447222
O	-2.081056	1.726993	-0.413439
C	-1.463446	2.909764	0.152687
Sn	2.092064	-0.149716	-0.422474
Cl	1.318281	-1.717060	-2.007932
Cl	1.507758	-0.458685	1.843571
Cl	2.642016	1.993609	-1.205007
H	-3.718781	0.546441	-1.818922
H	-3.447350	-1.097988	-2.592225
H	-1.226928	-1.264720	-1.535425
H	-0.699213	3.303136	-0.526223
H	-1.002300	2.675653	1.118717
H	-2.280975	3.624541	0.275275
C	-3.242032	0.177803	2.728305
H	-5.208794	0.034614	0.932913
Cl	4.304295	-0.997914	-0.246737
H	-2.236242	0.219648	3.161664

H	-3.926345	-0.187011	3.508188
H	-3.556784	1.198926	2.473442

P: 1,4_$SnCl_4$-ester-diene

E = −3683.66

H = −3534.76

***G* =** −3581.72

N_{imag} **=** 0

C	-3.263360	-0.431395	1.302182
C	-3.887849	-0.590629	0.125805
C	-3.303960	-0.044809	-1.157251
C	-1.935472	0.254339	1.484391
H	-4.102262	0.441045	-1.741521
H	-2.956693	-0.885906	-1.782888
H	-1.311270	-0.325934	2.181016
H	-2.076404	1.244177	1.949021
C	-2.163720	0.953899	-0.934500
C	-1.193236	0.411329	0.139497
C	0.030358	1.280873	0.264230
O	1.196034	0.955679	0.004072
O	-0.244018	2.518682	0.689197
C	0.886147	3.425356	0.819403
Sn	2.403198	-1.034135	-0.756383
Cl	1.001968	-0.774235	-2.629172
Cl	1.481382	-1.950561	1.203904
Cl	4.159923	0.490236	-0.510185
H	-2.566735	1.917775	-0.591817
H	-1.629151	1.134346	-1.876859
H	-0.836643	-0.576429	-0.181220
H	1.375985	3.557046	-0.151149
H	1.606990	3.026160	1.540678
H	0.454933	4.364382	1.172935
H	-3.725726	-0.832708	2.207889
C	-5.192403	-1.326876	-0.000140
H	-5.979878	-0.671598	-0.404702
H	-5.100621	-2.172755	-0.699850
H	-5.532892	-1.718803	0.966114
Cl	3.619645	-2.923913	-1.490771

P: 1,3_$SnCl_4$-ester-diene

E = −3681.29

H = −3533.03

***G* =** 3577.89

N_{imag} **=** 0

C	2.976445	-1.275442	-0.675985
C	3.659887	-0.145208	-0.446417
C	3.180364	0.963343	0.448111
C	1.629688	-1.547173	-0.045579
H	4.624526	-0.002457	-0.940858
H	2.847761	1.818485	-0.166069
H	4.020530	1.347499	1.047356

H	1.737320	-2.370085	0.681629
H	0.940493	-1.936121	-0.812187
C	2.064375	0.509960	1.393234
C	0.982019	-0.351187	0.688908
C	0.104010	0.500791	-0.197583
O	-1.008600	0.950490	0.109292
O	0.635158	0.778740	-1.390542
C	-0.166243	1.606617	-2.279585
Sn	-2.756614	0.626930	1.800956
Cl	-1.139407	1.565778	3.223642
Cl	-2.491393	-1.621250	1.156053
Cl	-3.837849	2.056362	0.298332
H	1.595272	1.364071	1.898310
H	0.303073	-0.734724	1.460171
H	-0.349977	2.583719	-1.820524
H	-1.123155	1.116774	-2.488001
H	0.432396	1.704396	-3.187687
C	3.498428	-2.367475	-1.568462
H	4.493749	-2.126309	-1.961236
H	3.566217	-3.324992	-1.027609
H	2.822881	-2.538757	-2.421667
H	2.495442	-0.126075	2.180251
Cl	-4.491547	0.332677	3.380003

TS: 1,4_$TiCl_4$-ester-diene
E = −3782.67
H = −3636.49
G **=** −3683.65
N_{imag} **=** 1,−338.858i cm^{-1}

C	-3.003684	-0.618404	1.566328
C	-4.174446	-0.534786	0.752854
C	-4.273133	-1.281785	-0.417947
C	-1.933740	-1.417332	1.299220
H	-5.193389	-1.217585	-0.999284
H	-3.763608	-2.241062	-0.484300
H	-1.977010	-2.227822	0.576187
H	-1.017387	-1.345131	1.883161
C	-2.951120	-0.400204	-1.740401
C	-1.626151	-0.480659	-1.312829
C	-0.910623	0.621097	-0.736394
O	0.326369	0.648669	-0.527088
O	-1.650865	1.714649	-0.459672
C	-0.930425	2.847442	0.090402
Ti	2.262693	-0.139801	-0.382674
Cl	1.663274	-1.681671	-1.897527
Cl	1.718592	-0.411395	1.787912
Cl	2.935566	1.842576	-1.158455
H	-3.426941	0.577596	-1.775504
H	-3.263890	-1.092640	-2.521392
H	-1.010316	-1.347033	-1.543666
H	-0.140724	3.168572	-0.597348

H	-0.484875	2.582386	1.055906
H	-1.683055	3.630345	0.211662
H	-2.920913	0.098680	2.387329
C	-5.191983	0.524970	1.077380
H	-4.733460	1.524481	1.107328
H	-6.014114	0.539960	0.352943
H	-5.621689	0.346458	2.074639
Cl	4.320431	-0.960826	-0.117802

TS: 1,3_TiCl$_4$-ester-diene

E = −3782.59

H = −3636.36

***G* =** −3681.97

N_{imag} **=** 1,−356.667i cm^{-1}

C	-2.931107	-0.685128	1.534636
C	-4.097881	-0.621424	0.721748
C	-4.247575	-1.305481	-0.476950
C	-1.876937	-1.483375	1.171459
H	-5.179371	-1.187072	-1.029755
H	-3.768221	-2.274713	-0.606458
H	-1.980682	-2.280244	0.440899
H	-0.928833	-1.443306	1.706819
C	-2.924154	-0.412809	-1.793314
C	-1.606066	-0.496506	-1.345621
C	-0.898732	0.610732	-0.760456
O	0.336173	0.648720	-0.548798
O	-1.652026	1.693392	-0.482763
C	-0.946049	2.837450	0.064037
Ti	2.291528	-0.118238	-0.427194
Cl	1.691686	-1.670935	-1.928678
Cl	1.772481	-0.390563	1.748548
Cl	2.922348	1.870173	-1.220088
H	-3.401553	0.563834	-1.821542
H	-3.226513	-1.098537	-2.584204
H	-0.980247	-1.352953	-1.585461
H	-0.165407	3.169537	-0.628736
H	-0.491187	2.579667	1.026918
H	-1.710392	3.608220	0.188808
C	-2.830189	0.241779	2.723996
H	-4.831398	0.151739	0.965776
Cl	4.362377	-0.907487	-0.184409
H	-1.817821	0.245390	3.143492
H	-3.520136	-0.079653	3.517836
H	-3.102678	1.271754	2.456291

P: 1,4_TiCl$_4$-ester-diene

E = −3830.44

H = −3681.22

***G* =** −3726.88

N_{imag} **=** 0

C	-3.288547	-0.426663	1.305098

C	-3.915413	-0.585581	0.129930
C	-3.322125	-0.061137	-1.157674
C	-1.949329	0.237902	1.482008
H	-4.111879	0.433121	-1.746493
H	-2.989221	-0.913420	-1.775912
H	-1.335192	-0.346670	2.183971
H	-2.073484	1.233886	1.938333
C	-2.165481	0.920406	-0.943823
C	-1.204258	0.370870	0.135891
C	0.032041	1.221282	0.251454
O	1.186853	0.869424	-0.020949
O	-0.210797	2.462908	0.677467
C	0.940224	3.345891	0.795209
Ti	2.394077	-0.875047	-0.716734
Cl	1.132857	-0.692792	-2.552665
Cl	1.599365	-1.842816	1.132352
Cl	4.096311	0.526117	-0.491571
H	-2.552289	1.893937	-0.609595
H	-1.627814	1.083544	-1.887474
H	-0.860351	-0.623969	-0.176758
H	1.421128	3.467124	-0.181108
H	1.659997	2.930504	1.508288
H	0.533009	4.293575	1.153779
H	-3.757726	-0.812272	2.214098
C	-5.232264	-1.300614	0.010063
H	-6.008438	-0.635571	-0.400335
H	-5.154827	-2.153952	-0.682277
H	-5.579434	-1.678282	0.979611
Cl	3.557279	-2.627066	-1.412258

P: 1,3_TiCl$_4$-ester-diene
E = −3828.54
H = −3680.03
***G* =** −3723.41
$\boldsymbol{N}_{\mathbf{imag}}$ **=** 0

C	3.090255	-1.141557	-0.744125
C	3.559356	-0.022722	-0.172274
C	2.846596	0.736807	0.913869
C	1.757586	-1.743064	-0.352557
H	4.520729	0.376846	-0.506465
H	2.442746	1.683233	0.510457
H	3.567544	1.043974	1.687164
H	1.936537	-2.673491	0.213419
H	1.218208	-2.054604	-1.259785
C	1.729378	-0.084007	1.564689
C	0.872321	-0.826127	0.507345
C	0.033599	0.164892	-0.266291
O	-0.888476	0.825931	0.229850
O	0.346549	0.300456	-1.552506
C	-0.448745	1.252280	-2.317494
Ti	-2.352780	1.328247	1.789781

Cl	-0.818687	2.860937	2.327087
Cl	-2.153593	-0.806689	2.425278
Cl	-3.719033	1.722632	0.080842
H	1.093275	0.548171	2.196048
H	0.126791	-1.426213	1.052207
H	-0.319557	2.259244	-1.907165
H	-1.506830	0.974026	-2.279195
H	-0.058253	1.190185	-3.335262
C	3.850018	-1.886150	-1.806362
H	4.819425	-1.417008	-2.014401
H	4.032652	-2.930191	-1.505107
H	3.277448	-1.928706	-2.746512
H	2.168172	-0.856264	2.212532
Cl	-3.777983	1.888307	3.391191

TS: 1,4_ZnCl$_2$-ester-diene

E = −3453.11

H = −3310.99

***G* =** −3353.59

$\boldsymbol{N_{imag}}$ **=** 1,−349.753i cm^{-1}

C	2.490236	-1.386925	-0.556912
C	3.496909	-0.735903	0.216192
C	3.307360	-0.527109	1.580908
C	1.322396	-1.873587	-0.049270
H	4.107886	-0.051137	2.148225
H	2.715791	-1.240295	2.151860
H	1.174936	-2.032843	1.016075
H	0.536111	-2.249253	-0.704438
C	1.852100	0.917255	1.717453
C	0.627512	0.503758	1.191614
C	0.122726	0.924791	-0.088482
O	-1.026663	0.689329	-0.537268
O	0.975277	1.675412	-0.819465
C	0.485000	2.116079	-2.109246
Zn	-2.334963	-0.752871	0.102097
Cl	-2.415685	-2.183418	-1.488518
Cl	-3.002996	-0.354535	2.095330
H	2.394420	1.707788	1.202862
H	1.945815	0.928053	2.803012
H	-0.095006	-0.021477	1.815738
H	-0.384705	2.770717	-1.983172
H	0.203267	1.256027	-2.727085
H	1.317207	2.664155	-2.558095
H	2.618070	-1.380065	-1.642664
C	4.648585	-0.092724	-0.508376
H	4.296812	0.629157	-1.260455
H	5.327748	0.424345	0.178950
H	5.229624	-0.853636	-1.050835

TS: 1,3_$ZnCl_2$-ester-diene
E = −3452.14
H = −3309.97
***G* =** −3351.99
N_{imag} = 1,−365.987i cm^{-1}

C	2.439091	-1.444401	-0.541569
C	3.443790	-0.809972	0.240082
C	3.311996	-0.537890	1.596042
C	1.272397	-1.869206	0.043305
H	4.135968	-0.039989	2.107183
H	2.739489	-1.217063	2.226290
H	1.184856	-2.009569	1.116901
H	0.444575	-2.243185	-0.560810
C	1.857002	0.913653	1.734079
C	0.640675	0.490170	1.197807
C	0.144877	0.918728	-0.087192
O	-1.003741	0.696736	-0.541319
O	1.009997	1.660981	-0.808939
C	0.530420	2.122315	-2.096236
Zn	-2.346195	-0.721505	0.089660
Cl	-2.468900	-2.126903	-1.519467
Cl	-2.989072	-0.323057	2.090495
H	2.402827	1.700212	1.218365
H	1.943315	0.927441	2.820142
H	-0.092704	-0.023493	1.818487
H	-0.326374	2.792490	-1.964048
H	0.232647	1.273661	-2.721834
H	1.374122	2.657841	-2.538397
C	2.626836	-1.517341	-2.039510
H	4.273345	-0.344391	-0.298825
H	2.934814	-0.548201	-2.455176
H	1.707884	-1.838816	-2.542560
H	3.415453	-2.241887	-2.289795

P: 1,4_$ZnCl_2$-ester-diene
E = −3500.76
H = −3356.26
***G* =** −3395.57
N_{imag} = 0

C	-2.947992	-0.758925	1.070123
C	-3.727448	-0.658405	-0.016610
C	-3.380424	0.276674	-1.152982
C	-1.657524	-0.010663	1.273903
H	-4.291256	0.804431	-1.478439
H	-3.062388	-0.320251	-2.026021
H	-0.895026	-0.684932	1.693284
H	-1.796311	0.789891	2.021929
C	-2.299495	1.301256	-0.789409
C	-1.145436	0.601796	-0.056386
C	0.048299	1.473726	0.230971
O	1.211353	1.062592	0.350803

O	-0.239759	2.764508	0.400542
C	0.875730	3.637805	0.735680
Zn	1.999912	-0.879752	0.065316
H	-5.147913	-2.142204	0.666675
Cl	2.742544	-1.461324	1.966965
Cl	1.647755	-1.333271	-1.987322
H	-2.724942	2.077577	-0.137590
H	-1.932957	1.804700	-1.694038
H	-0.765934	-0.219234	-0.686410
H	1.615780	3.625291	-0.071514
H	1.343771	3.307144	1.668859
H	0.433540	4.630085	0.846377
H	-3.240829	-1.435317	1.877353
C	-4.982191	-1.469051	-0.183260
H	-5.863747	-0.816794	-0.285827
H	-4.936706	-2.078725	-1.099526

P: 1,3_$ZnCl_2$-ester-diene

E = −3499.72

H = −3354.72

***G* =** −3395.48

N_{imag} **=** 0

C	3.057040	-1.162565	-0.754656
C	3.564029	-0.034628	-0.234991
C	2.895355	0.780838	0.839165
C	1.719723	-1.716380	-0.311971
H	4.526665	0.330113	-0.603470
H	2.512469	1.726199	0.413901
H	3.639375	1.089162	1.589619
H	1.887920	-2.621309	0.296661
H	1.156392	-2.059071	-1.192379
C	1.765772	0.013647	1.533477
C	0.872693	-0.736576	0.514579
C	0.057626	0.252308	-0.291196
O	-0.759728	1.040525	0.208608
O	0.260506	0.229702	-1.606001
C	-0.528042	1.162029	-2.398909
Zn	-1.541548	1.121295	2.165844
Cl	-1.219587	3.104203	2.851473
Cl	-2.304564	-0.839168	2.532096
H	2.188831	-0.745244	2.206459
H	1.166920	0.692454	2.157922
H	0.108162	-1.292673	1.083771
H	-0.327492	2.189559	-2.078258
H	-1.594624	0.941947	-2.283119
H	-0.203156	1.000335	-3.428729
C	3.776595	-1.964152	-1.803180
H	4.751290	-1.525834	-2.050009
H	3.942641	-2.999574	-1.465055

H	3.182168	-2.030009	-2.728210

TS: 1,4_BF$_3$-ester-diene
E = −3823.75
H = −3675.53
G **=** −3716.25
N_{imag} **=** 1,-338.110i cm^{-1}

C	-1.964540	-0.331341	1.537797
C	-2.965330	-0.518989	0.536091
C	-2.799579	-1.482555	-0.454791
C	-0.816330	-1.057920	1.625488
H	-2.080921	0.535835	2.193486
H	-3.597694	-1.619402	-1.185018
H	-2.227935	-2.382211	-0.235576
H	-0.669361	-1.992768	1.091821
H	-0.024510	-0.789685	2.321845
C	-1.307137	-0.743802	-1.682725
C	-0.079869	-0.644525	-1.028360
C	0.460932	0.594940	-0.560419
O	1.634168	0.786612	-0.120145
O	-0.351631	1.661475	-0.649093
C	0.200239	2.930443	-0.215786
H	-1.813750	0.170200	-1.985341
H	-1.441204	-1.588963	-2.356936
H	0.597514	-1.492326	-0.972059
H	1.084087	3.184617	-0.810736
H	0.478515	2.884637	0.843096
H	-0.600308	3.656245	-0.378058
C	-4.087573	0.480435	0.456181
H	-3.704620	1.508100	0.370248
H	-4.752192	0.283857	-0.392726
H	-4.692353	0.446452	1.375039
B	2.789698	-0.358220	0.056425
F	3.854482	0.357205	0.536558
F	2.270552	-1.274782	0.964006
F	2.983466	-0.900651	-1.201910

TS: 1,3_BF$_3$-ester-diene
E = −3822.62
H = −3674.36
G **=** −3714.61
N_{imag} **=** 1,-354.779i cm^{-1}

C	-1.922265	-0.326163	1.578292
C	-2.916661	-0.553030	0.584720
C	-2.801634	-1.491948	-0.430930
C	-0.775709	-1.076840	1.593376
C	-2.099823	0.845273	2.516939
H	-3.618767	-1.586230	-1.145939
H	-2.250946	-2.414179	-0.251598
H	-0.690722	-2.018711	1.060165
H	0.058957	-0.834618	2.248840

C	-1.305237	-0.777622	-1.672833
C	-0.087662	-0.664889	-1.002948
C	0.446999	0.590893	-0.561575
O	1.621609	0.801704	-0.138258
O	-0.378182	1.644552	-0.664163
C	0.167245	2.932086	-0.277402
H	-1.811751	0.129943	-1.992540
H	-1.430414	-1.634434	-2.333877
H	0.598578	-1.504482	-0.934888
H	1.037695	3.176411	-0.895666
H	0.464332	2.920617	0.777013
H	-0.644177	3.643779	-0.447225
H	-3.724198	0.180880	0.514103
H	-1.201671	1.012588	3.122276
H	-2.936902	0.660333	3.205704
H	-2.330436	1.769635	1.969496
B	2.804338	-0.324860	0.018943
F	3.863534	0.413170	0.474588
F	2.319791	-1.246824	0.938499
F	2.978700	-0.864172	-1.242779

P: 1,4_BF_3-ester-diene
E = −3870.58
H = −3719.32
***G* =** −3758.45
N_{imag} **=** 0

C	-3.256888	-0.396275	1.321147
C	-3.830490	-0.609879	0.127472
C	-3.198711	-0.112537	-1.152189
C	-1.943133	0.309593	1.529422
H	-3.975342	0.342503	-1.788287
H	-2.817013	-0.973793	-1.727532
H	-1.346338	-0.235555	2.277075
H	-2.111814	1.314882	1.948105
C	-2.076628	0.905465	-0.927743
C	-1.148852	0.410043	0.212967
C	0.056225	1.298991	0.308061
O	1.185160	1.065622	-0.171616
O	-0.150232	2.450264	0.934192
C	0.976639	3.372388	1.007975
B	1.667488	-0.349837	-1.006321
F	0.810226	-0.408482	-2.081266
F	1.490963	-1.353862	-0.084091
F	2.960280	-0.052110	-1.307334
H	-2.500270	1.879367	-0.640523
H	-1.500816	1.043428	-1.851423
H	-0.775453	-0.584558	-0.060994
H	1.296941	3.651346	-0.001133
H	1.808892	2.901584	1.541110
H	0.594707	4.236554	1.555073
H	-3.753284	-0.765096	2.222549

C	-5.122041	-1.364598	-0.021314
H	-5.897788	-0.735397	-0.485442
H	-4.992874	-2.237611	-0.680574
H	-5.499061	-1.719822	0.945595

P: 1,3_BF_3-ester-diene

E = −3867.79

H = −3716.59

***G* =** −3755.58

***N*$_{imag}$ =** 0

C	2.527852	-1.396275	-0.448971
C	3.529420	-0.527063	-0.651230
C	3.695755	0.776169	0.079558
C	1.428063	-1.133432	0.554674
H	4.291052	-0.767920	-1.398337
H	3.589399	1.611366	-0.631057
H	4.726768	0.850855	0.461238
H	1.606594	-1.731559	1.463627
H	0.464334	-1.486765	0.163552
C	2.716416	0.940563	1.252738
C	1.319377	0.354913	0.985767
C	0.466726	1.131490	0.012508
O	-0.753164	0.957205	-0.185955
O	1.088329	2.087347	-0.663439
C	0.281075	2.865950	-1.596044
B	-1.920632	-0.033534	0.592733
F	-1.509506	-0.116243	1.903347
F	-1.847638	-1.210067	-0.106976
F	-3.048839	0.700640	0.390360
H	2.644788	1.995528	1.548855
H	0.725085	0.373788	1.911303
H	-0.538064	3.356728	-1.060866
H	-0.126688	2.209581	-2.371463
H	0.972677	3.596560	-2.020178
C	2.431994	-2.705906	-1.182360
H	3.278910	-2.849616	-1.864598
H	2.412977	-3.551860	-0.476813
H	1.499455	-2.767529	-1.764324
H	3.108815	0.396605	2.124409

TS: 1,4_$AlCl_3$-ester-diene

E = −3640.05

H = −3495.36

***G* =** −3538.56

***N*$_{imag}$ =** 1,−314.503i cm^{-1}

C	2.507402	0.025912	1.555402
C	3.647208	0.258053	0.721087
C	3.690838	1.352773	-0.135039
C	1.411041	0.828536	1.611907
H	4.594672	1.519325	-0.721503
H	3.139319	2.255817	0.118426

H	1.392005	1.825532	1.178095
H	0.524795	0.537660	2.175806
C	2.365201	0.884846	-1.670736
C	1.048193	0.798422	-1.223785
C	0.375882	-0.437954	-0.994125
O	-0.860262	-0.563730	-0.728230
O	1.105112	-1.552664	-1.140991
C	0.417531	-2.814974	-0.930608
Al	-2.310594	0.341992	-0.042143
Cl	-2.000685	2.441420	-0.352738
Cl	-2.234284	-0.194197	2.031983
Cl	-3.957636	-0.462728	-1.112293
H	2.864435	-0.019435	-2.012335
H	2.656372	1.797483	-2.189246
H	0.419588	1.682908	-1.131031
H	-0.400990	-2.925514	-1.649713
H	0.018812	-2.865626	0.088437
H	1.180871	-3.579904	-1.090481
H	2.482914	-0.921363	2.100712
C	4.708001	-0.806462	0.660650
H	4.275792	-1.793041	0.439025
H	5.474019	-0.581434	-0.089968
H	5.207706	-0.895916	1.637485

TS: 1,3_$AlCl_3$-ester-diene

E = −3638.87

H = −3494.11

***G* =** −3536.86

N_{imag} **=** 1,−356.648i cm^{-1}

C	2.641499	0.651278	-1.243841
C	3.759041	0.061226	-0.582901
C	3.863902	-1.286139	-0.271090
C	1.584819	-0.121594	-1.646251
H	4.492467	0.744777	-0.146220
H	4.768388	-1.635204	0.226484
H	3.382828	-2.026693	-0.908169
H	1.649351	-1.204212	-1.709453
H	0.670218	0.326215	-2.036396
C	2.483824	-1.605993	1.245174
C	1.184328	-1.324335	0.826566
C	0.493949	-0.122834	1.176203
O	-0.736921	0.106286	0.966818
O	1.203792	0.797694	1.840279
C	0.499694	2.005523	2.237214
Al	-2.180198	-0.424274	-0.051919
Cl	-1.860709	-2.456159	-0.655755
Cl	-2.093525	0.943828	-1.699402
Cl	-3.833784	-0.150352	1.250233
H	2.945900	-0.960435	1.988361
H	0.571864	-2.070412	0.322547
H	-0.328681	1.756359	2.908697

H	0.112220	2.527430	1.355781
H	1.249810	2.610079	2.751942
C	2.600603	2.155848	-1.379732
H	2.839946	2.653333	-0.429855
H	3.344808	2.492269	-2.116286
H	1.616953	2.499617	-1.718563
H	2.774297	-2.654597	1.297406

P: 1,4_AlCl$_3$-ester-diene
E = −3684.91
H = −3537.21
***G* =** −3578.98
***N*$_{imag}$ =** 0

C	-3.338845	-0.338643	1.329304
C	-3.887896	-0.588623	0.131257
C	-3.228441	-0.134016	-1.150017
C	-2.027334	0.369464	1.543106
H	-3.989110	0.305802	-1.815187
H	-2.839297	-1.013189	-1.691927
H	-1.447469	-0.153497	2.319083
H	-2.198788	1.389584	1.923261
C	-2.107449	0.887025	-0.936772
C	-1.205681	0.424341	0.240584
C	-0.000398	1.307892	0.327617
O	1.134179	1.030725	-0.127864
O	-0.196133	2.480965	0.897635
C	0.941558	3.396609	0.957500
Al	1.959716	-0.454621	-0.957901
Cl	0.882478	-0.671963	-2.786156
Cl	1.647711	-2.039806	0.433086
Cl	3.961193	0.209479	-1.155162
H	-2.530191	1.871515	-0.687621
H	-1.513102	0.991423	-1.853666
H	-0.830358	-0.583126	0.008571
H	1.279226	3.633909	-0.056188
H	1.757554	2.936103	1.523000
H	0.556993	4.282684	1.465539
H	-3.852787	-0.679039	2.231746
C	-5.177321	-1.345844	-0.020860
H	-5.941312	-0.730605	-0.521514
H	-5.035392	-2.239536	-0.648810
H	-5.575566	-1.669299	0.948527

P: 1,3_AlCl$_3$-ester-diene
E = −3682.86
H = −3535.24
***G* =** −3576.71
***N*$_{imag}$ =** 0

C	2.593197	-1.405901	-0.416670
C	3.583287	-0.530536	-0.648160
C	3.747983	0.787862	0.056104

C	1.500498	-1.130169	0.591740
H	4.338840	-0.779876	-1.398357
H	3.621408	1.610347	-0.666462
H	4.784123	0.880518	0.418619
H	1.682204	-1.711247	1.510424
H	0.536361	-1.496421	0.208011
C	2.787109	0.961073	1.242938
C	1.392801	0.365703	0.992378
C	0.534438	1.117844	0.012570
O	-0.671591	0.858270	-0.207801
O	1.099419	2.123185	-0.622733
C	0.262438	2.885838	-1.549534
Al	-2.175313	-0.046645	0.464414
Cl	-1.791565	-0.256410	2.555842
Cl	-2.188259	-1.872089	-0.626699
Cl	-3.735642	1.312979	-0.004765
H	2.712983	2.018332	1.529316
H	0.793177	0.401317	1.919163
H	-0.606880	3.287646	-1.020290
H	-0.065996	2.235084	-2.365867
H	0.911750	3.683052	-1.915423
C	2.501914	-2.732957	-1.118008
H	3.339727	-2.881392	-1.810121
H	2.505481	-3.562021	-0.392651
H	1.560772	-2.819819	-1.682138
H	3.192345	0.427200	2.114519
